# High dietary potassium causes ubiquitin-dependent degradation of the kidney sodium-chloride cotransporter

**DOI:** 10.1016/j.jbc.2021.100915

**Published:** 2021-06-24

**Authors:** Marleen L.A. Kortenoeven, Cristina Esteva-Font, Henrik Dimke, Søren B. Poulsen, Sathish K. Murali, Robert A. Fenton

**Affiliations:** 1 Department of Biomedicine, Faculty of Health Sciences, Aarhus University, Aarhus, Denmark; 2 Department of Cardiovascular and Renal Research, Institute of Molecular Medicine, University of Southern Denmark, Odense, Denmark; 3 Department of Nephrology, Odense University Hospital, Odense, Denmark

**Keywords:** posttranslational modification (PTM), kidney, hypertension, aldosterone, ubiquitylation (ubiquitination), BP, blood pressure, CHIP, C-terminus of Hsc70 interacting protein, DCT, distal convoluted tubule, Hsp70, heat shock protein 70, NCC, sodium-chloride cotransporter, PHAII, pseudohypoaldosteronism type II, PP1α, protein phosphatase 1α

## Abstract

The thiazide-sensitive sodium-chloride cotransporter (NCC) in the renal distal convoluted tubule (DCT) plays a critical role in regulating blood pressure (BP) and K^+^ homeostasis. During hyperkalemia, reduced NCC phosphorylation and total NCC abundance facilitate downstream electrogenic K^+^ secretion and BP reduction. However, the mechanism for the K^+^-dependent reduction in total NCC levels is unknown. Here, we show that NCC levels were reduced in *ex vivo* renal tubules incubated in a high-K^+^ medium for 24–48 h. This reduction was independent of NCC transcription, but was prevented using inhibitors of the proteasome (MG132) or lysosome (chloroquine). *Ex vivo*, high K^+^ increased NCC ubiquitylation, but inhibition of the ubiquitin conjugation pathway prevented the high K^+^-mediated reduction in NCC protein. In tubules incubated in high K^+^ media *ex vivo* or in the renal cortex of mice fed a high K^+^ diet for 4 days, the abundance and phosphorylation of heat shock protein 70 (Hsp70), a key regulator of ubiquitin-dependent protein degradation and protein folding, were decreased. Conversely, in similar samples the expression of PP1α, known to dephosphorylate Hsp70, was also increased. NCC coimmunoprecipitated with Hsp70 and PP1α, and inhibiting their actions prevented the high K^+^-mediated reduction in total NCC levels. In conclusion, we show that hyperkalemia drives NCC ubiquitylation and degradation *via* a PP1α-dependent process facilitated by Hsp70. This mechanism facilitates K^+^-dependent reductions in NCC to protect plasma K^+^ homeostasis and potentially reduces BP.

Hypertension is a worldwide public-health challenge because of its high frequency and concomitant risks of cardiovascular or kidney disease (1). The kidney's ability to adjust NaCl excretion plays a critical role in blood pressure (BP) control (2). The thiazide-sensitive sodium-chloride cotransporter (NCC), expressed in the distal convoluted tubule (DCT), is essential for BP control. This is highlighted by loss-of-function NCC mutations underlying hypotensive Gitelman's syndrome or activation of NCC in hypertensive pseudohypoaldosteronism type II (PHAII or Gordon syndrome) (3, 4, 5). Dietary K^+^ intake inversely associates with BP, with low dietary K^+^ intake increasing the risk of death and cardiovascular events, and a high dietary K^+^ intake associated with lower BP (6, 7, 8). The effects of K^+^ on BP are blunted in NCC knockout mice, highlighting that NCC plays an essential role in the antihypertensive effects of dietary K^+^ (9). Patients with Gitelman's syndrome suffer from hypokalemia, while patients with PHAII suffer from hyperkalemia, demonstrating that NCC is also essential for K^+^ homeostasis (3, 4).

During hyperkalemia, a reduction in both NCC phosphorylation (active form) and total NCC abundance enhances Na^+^ delivery to downstream segments of the renal tubule to facilitate electrogenic K^+^ secretion and restore plasma K^+^ levels. Similar reductions in total and phosphorylated NCC levels are further linked with the ability of a high K^+^ diet to lower BP. Reduced NCC phosphorylation following high dietary K^+^ intake can be explained by alterations in the basolateral plasma membrane potential *via* the inwardly rectifying potassium channel Kir4.1/Kir5.1 (a heterotetramer of Kir4.1 and Kir5.1 channels) and modulation of the WNK-SPAK/OSR1 kinase signaling pathway (10, 11, 12, 13). However, such a mechanism cannot easily account for a sustained reduction in total NCC following high dietary K^+^ intake. Therefore, the aim of this study was to identify the mechanism of how high K^+^ decreases total NCC abundance. Our findings suggest that high K^+^ increases ubiquitin-dependent NCC degradation in a mechanism facilitated by protein phosphatase 1α (PP1α) effects on heat shock protein 70. This mechanism helps increase K^+^ secretion during hyperkalemia and is a novel concept for understanding how high dietary K^+^ can reduce BP.

## Results

### Long-term high K^+^ exposure reduces NCC abundance in renal cortical tubules

**Figure 1:**
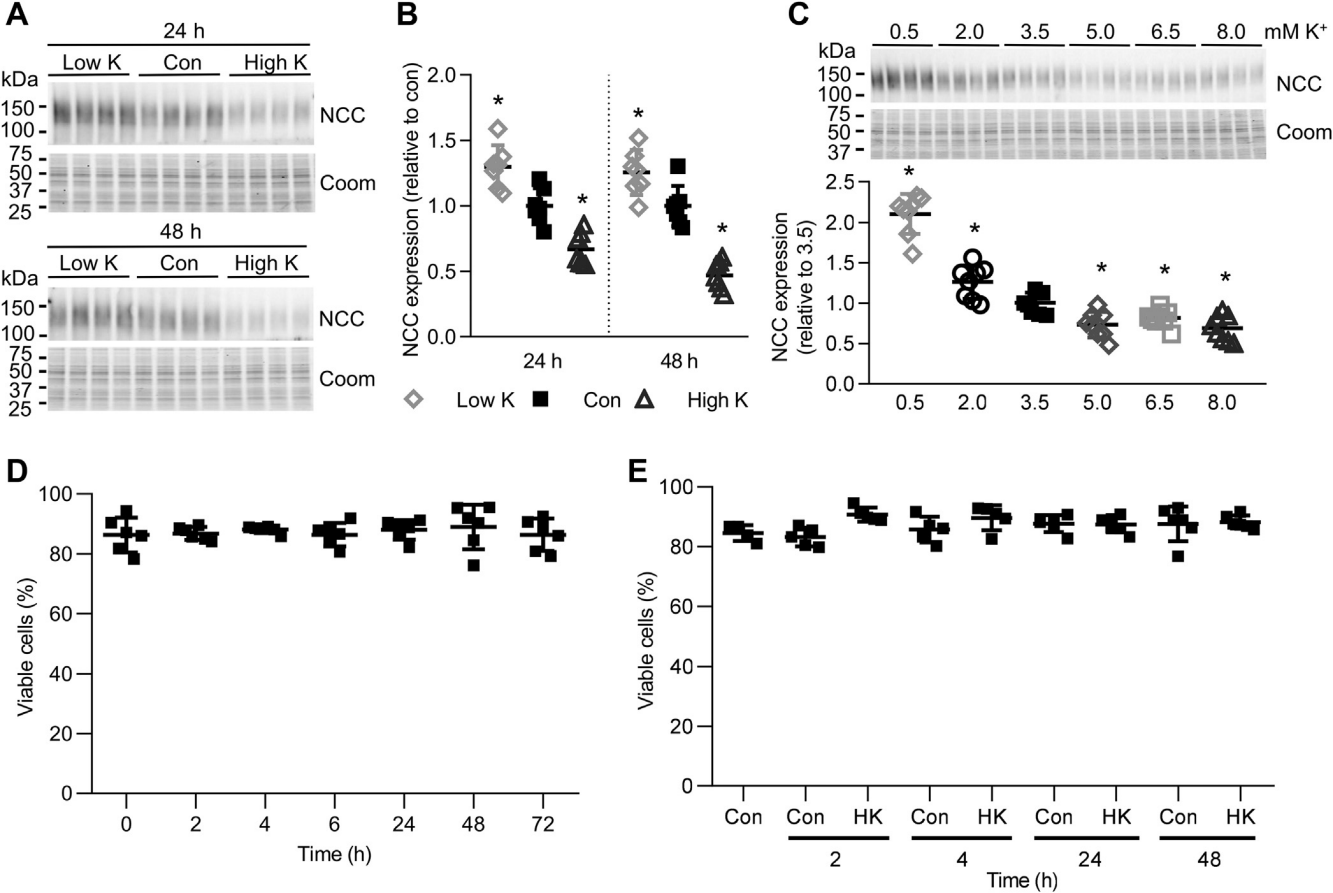
**High K**^**+**^**decreases NCC abundance**. *A*, isolated renal cortical tubules were incubated in either low K^+^ (0.5 mM), control (Con, 3.5 mM) or high K^+^ (8.0 mM) medium and NCC protein levels assessed after 24 or 48 h. *B*, summary data of normalized band densities relative to control (n = 7). ∗*p* < 0.05 compared with control. *C*, tubules were incubated in medium with varying K^+^ concentrations and NCC protein levels assessed after 24 h. Summary data of NCC normalized band densities relative to 3.5 mM K^+^ control media (mean ± SD, n = 8). ∗*p* < 0.05 compared with control media. *D*, tubules were incubated in control medium, and the number of viable cells was measured 0–72 h after seeding. n = 6. *E*, tubules were incubated in either control (3.5 mM) or high K (8.0 mM) medium, and the number of viable cells was measured 0–48 h after seeding. n = 5. *B–E*, shown are mean values ± SD. Comparisons were performed using a one-way ANOVA followed by a Dunnett multiple comparison test. Coom, Coomassie blue staining.

Aldosterone increases renal NCC expression and its phosphorylation, while total renal NCC expression and phosphorylation are lower under a high-K^+^ diet, even though aldosterone levels are increased (14, 15, 16). It is thought that this reduction in NCC is triggered by an increase in plasma K^+^ subsequent to the high-K^+^ diet (9). To assess the ability of long-term alterations in K^+^ to modulate NCC abundance independently of aldosterone, we developed an *ex vivo* system that utilizes renal cortical tubules isolated from mice. Incubation of such preparations in different concentrations of KCl for 24 or 48 h (choline Cl was used to balance osmolality and chloride when adjusting K^+^ levels) demonstrated that relative to control conditions (3.5 mM K^+^), NCC levels were significantly higher after incubation in 0.5 mM K^+^ and significantly decreased in 8 mM K^+^ media (Fig. 1, *A* and *B*). Similar experiments balancing altered KCl concentrations with NaCl (instead of choline Cl) showed similar results (Fig. S1*A*). A K^+^ concentration curve confirmed the effects on NCC after 24 h occurred within the physiological range, with NCC levels significantly increased in K^+^ concentrations lower than 3.5 mM, but decreased in K^+^ concentrations greater than 3.5 mM (Fig. 1*C*).

To investigate if the effect of K^+^ is affected by the accompanying anion, similar tubule experiments were performed using either KCl, K-citrate, or Na-citrate. KCl and K-citrate significantly decreased the expression of NCC (Fig. S1*B*), indicating that the effects of K^+^ in this setting are independent of the anion. To rule out that the effects of high K^+^ to reduce NCC abundance are not due to cell death during prolonged incubation of renal tubules or cytotoxic effects of the high K^+^, the viability of the renal tubule suspensions was investigated. No decrease in cell number, viability, or mitochondrial metabolic activity was observed in the tubules throughout the experimental period (up to 72 h) when incubated in either control or high K^+^ medium (Fig 1, *D* and *E* and Fig. S2).

**Figure 2:**
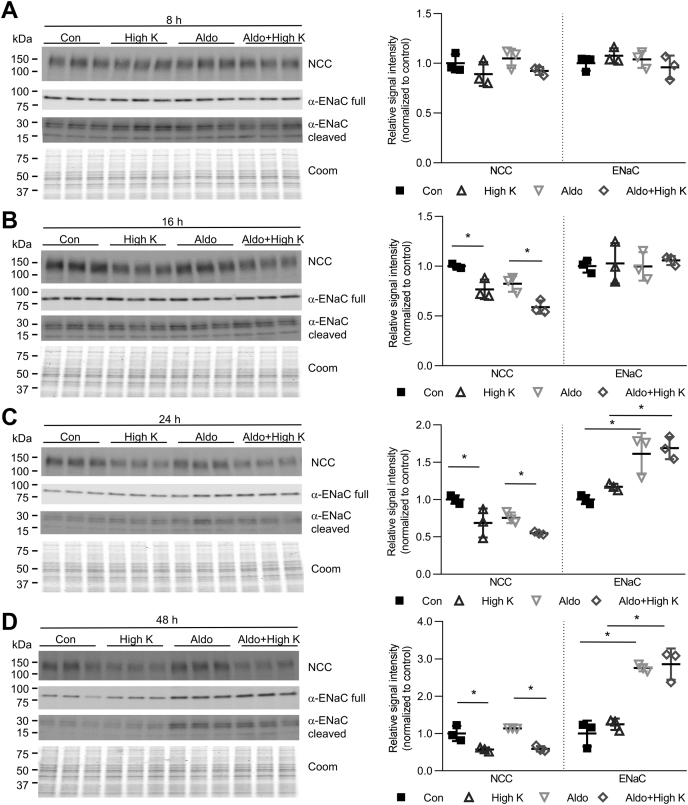
**High K**^**+**^**effects on NCC occur in presence of aldosterone**. Tubules were incubated in either control (Con, 3.5 mM K^+^) or high K^+^ (8.0 mM K^+^) medium with or without 10 nM aldosterone. Tubules were harvested after (*A*) 8 h, (*B*) 16 h, (*C*) 24 h, and (*D*) 48 h and subjected to immunoblotting for NCC or α-ENaC. Summary data (mean ± SD) show normalized signal intensity relative to control (n = 3) and significant differences are indicated (∗*p* < 0.05). Comparisons were performed using a two-way ANOVA followed by a Tukey multiple comparison test. Coom, Coomassie blue staining.

High plasma K^+^ will trigger the release of aldosterone from the adrenal gland. To assess if the effects of high K^+^ on NCC expression were still apparent with aldosterone present, tubules were incubated in high K^+^ medium with or without 10 nM aldosterone for 8–48 h. At 16–48 h, high K^+^ significantly decreased NCC, independent of the presence of 10 nM aldosterone. In contrast, α-ENaC abundance increased significantly after 24 h aldosterone incubation, with or without high K^+^ (Fig. 2).

Recently K^+^ has been shown to increase ENaC activity independently from aldosterone (17). To investigate if ENaC abundance is regulated by K^+^ in the cortical tubules, suspensions were incubated in low or high K^+^ medium for 24 or 48 h. Relative to control (3.5 mM K^+^), low K^+^ medium significantly decreased the protein expression of α-ENaC at both time points, while high K^+^ medium had no significant effect (Fig. S3).

### In renal cortical tubules high K^+^ drives ubiquitin-mediated degradation of NCC

**Figure 3:**
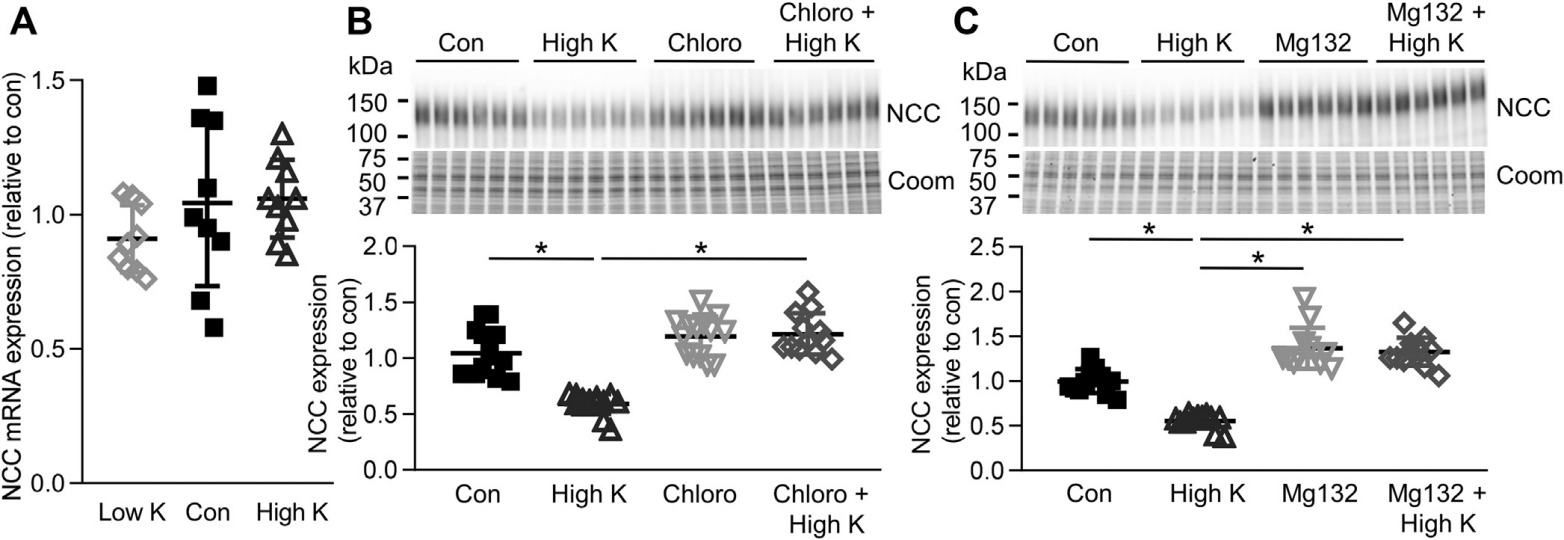
**High K**^**+**^**causes NCC degradation**. *A*, tubules were incubated in either low K^+^ (0.5 mM), control (Con, 3.5 mM), or high K^+^ (8.0 mM) medium and NCC mRNA levels assessed after 24 h using RTqPCR. Signals are normalized against 18S and expressed relative to control (mean values ± SD, n = 9). Comparisons were performed using a one-way ANOVA followed by a Dunnett multiple comparison test. *B* and *C*, tubules were incubated in control (3.5 mM) or high K^+^ medium (8.0 mM) with or without 100 μM of the lysosomal inhibitor chloroquine (*B*) or 10 μM of the proteosomal inhibitor Mg132 (*C*) and after 24 h NCC protein levels were assessed (n = 12). Significant differences are indicated (∗*p* < 0.05). Shown are mean values ± SD. Comparisons were performed using a two-way ANOVA followed by a Tukey multiple comparison test. Coom, Coomassie blue staining.

**Figure 4:**
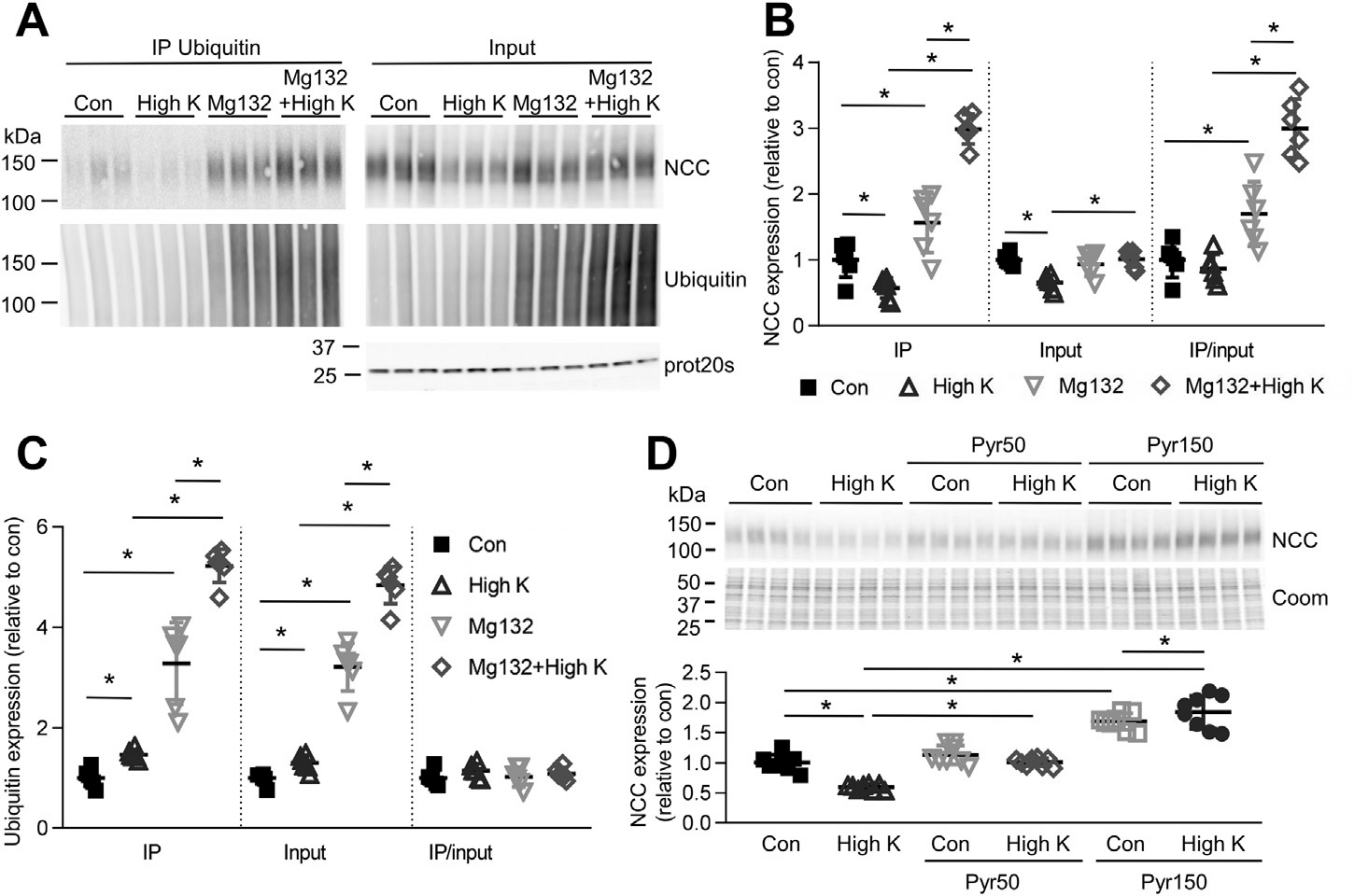
**High K**^**+**^**increases NCC ubiquitylation.***A*, isolated renal cortical tubules were incubated in control (3.5 mM) or high K^+^ medium (8.0 mM) with or without 10 μM of the proteosomal inhibitor Mg132. Lysates were subjected to immunoprecipitation using a ubiquitin antibody matrix. Immunoprecipitated samples and input samples were assessed for levels of NCC and ubiquitin. *B* and *C*, normalized band densities of NCC (*B*) and ubiquitin (*C*) relative to control (n = 6) Significant differences are indicated (∗*p* < 0.05). *D*, tubules were incubated in control (3.5 mM) or high K^+^ medium (8.0 mM) with or without Pyr-41, a blocker of the ubiquitin-activating E1 enzyme and after 24 h NCC protein levels were assessed. Significant differences are indicated (∗*p* < 0.05) (n = 8). *B–D*, shown are mean values ± SD. Comparisons were performed using a two-way ANOVA followed by a Tukey multiple comparison test. Coom, Coomassie blue staining; IP, immunoprecipitation.

No differences were detected in NCC mRNA expression after incubation of cortical tubules for 24 h in low or high K^+^ (Fig. 3*A*), suggesting that the observed effect of K^+^ to alter NCC levels was likely independent of changes in NCC transcription and may be due to enhanced NCC degradation. Supporting such a mechanism, the effects of high K^+^ on NCC were absent when tubules were simultaneously incubated for 24 h with the lysosomal inhibitor chloroquine or the proteasomal inhibitor MG132 (Fig. 3, *B* and *C*). As long-term incubation with MG132 can also reduce the degradation of proteins *via* lysosomes (18), caution must be exercised in concluding what percentage of high K^+^-induced NCC degradation occurs *via* the lysosomal or proteasomal pathways. The posttranslational modification ubiquitylation plays an important role in proteasomal and lysosomal protein degradation, and NCC can itself be ubiquitylated (19). Emphasizing a potential general role of K^+^ to modulate protein ubiquitylation, tubules incubated for 24 or 48 h in 0.5 mM K^+^ media had decreased protein ubiquitylation, whereas tubules incubated in 8 mM K^+^ had increased cellular ubiquitylation levels (Fig. S4*A*). To assess if K^+^ alters the quantity of ubiquitylated NCC, tubules incubated in high K^+^ medium with or without MG132 to block NCC degradation were immunoprecipitated using a ubiquitin antibody, and NCC and ubiquitin levels were analyzed. In line with a role of K^+^ to modulate protein ubiquitylation, high K^+^ alone led to a small increase in the total amount of ubiquitylated proteins, whereas high K^+^ in the presence of MG132 greatly increased the amount of ubiquitylated proteins compared with MG132 alone (Fig. 4, *A* and *C*). High K^+^ alone did not significantly change the amount of ubiquitylated NCC isolated (Fig. 4, *A* and *B*), probably as NCC is rapidly degraded once ubiquitylated. However, in the presence of MG132 to limit NCC degradation, high K^+^ significantly increased the abundance of ubiquitylated NCC compared with MG132 alone (Fig. 4, *A* and *B*). Immunoprecipitation using an NCC antibody gave similar results (not shown). To link increased ubiquitylation to the high K^+^-mediated reduction in total NCC levels, cortical tubules were incubated for 24 h in control or high K^+^ media with Pyr-41, a blocker of the ubiquitin-activating enzyme E1 (20). In total, 50 μM and 150 μM Pyr-41 prevented the decrease in NCC with high K^+^, with the higher dose increasing NCC levels under control and high K^+^ conditions (Fig. 4*D*). Taken together, this data demonstrates that high K^+^ reduces total NCC levels *via* an increase in ubiquitin-mediated degradation.

### A role for heat shock protein 70 in mediating the long-term effects of high K^+^ on NCC abundance

The balance between ubiquitin-dependent degradation and protein folding is maintained by the chaperone proteins heat shock protein 70 (Hsp70) and Hsp90 (21). In our recent large-scale proteomics study, the abundance of these and other heat shock proteins was increased specifically in the mouse DCT after a high dietary K^+^ intake (22), suggesting they play an important role in modulating NCC levels following a high K^+^ diet. The role of Hsp70 and Hsp90 depends on their interaction with other proteins including the C-terminus of Hsc70 interacting protein (CHIP, encoded by *Stub1*) and the Hsp70-Hsp90 organizing protein HOP (*Stip1*). If Hsp70-bound client proteins are bound to the cochaperone CHIP, this enhances degradation of the client protein (23).

**Figure 5:**
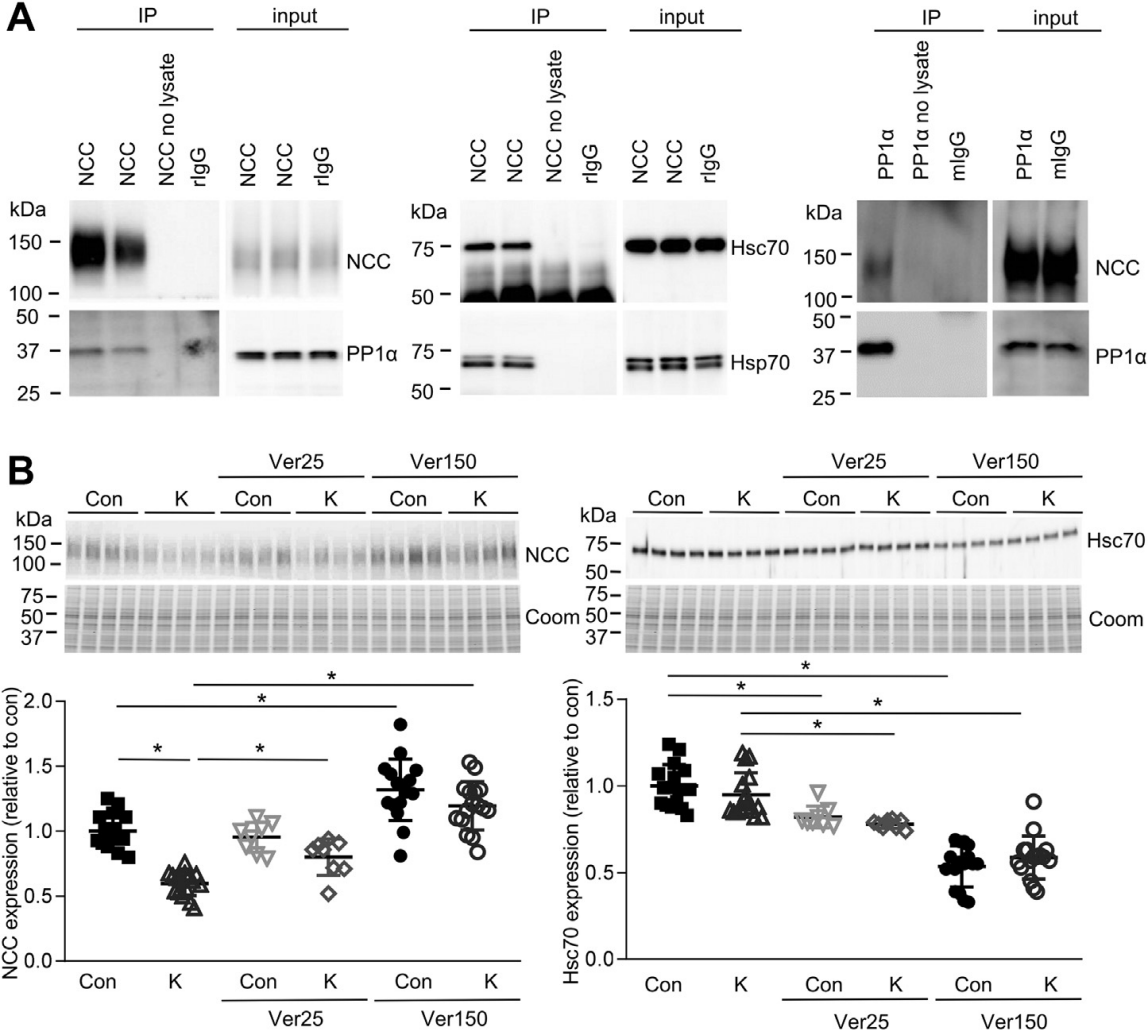
**Heat shock proteins are involved in the high K**^**+**^**-induced reduction in total NCC levels**. *A*, coimmunoprecipitation (IP) of NCC, PP1α, Hsc70, and Hsp70 from lysates isolated from cortical tubules grown in high K^+^ medium for 24 h. *B*, tubules were incubated in control (Con, 3.5 mM) or high K^+^ medium (K, 8.0 mM) for 24 h, with or without 25 or 150 μM of the Hsp70/Hsc70 inhibitor Ver-155008 (Ver), and NCC and Hsc70 levels assessed by immunoblotting. The same samples were used for blotting with NCC and Hsc70, hence the Coomassie gels for each panel are the same. Summary data (mean ± SD) show normalized band densities relative to control and significant differences are indicated (∗*p* < 0.05, n = 8–16). Comparisons were performed using a two-way ANOVA followed by a Tukey multiple comparison test. Coom, Coomassie blue staining; IP, immunoprecipitation.

**Figure 6:**
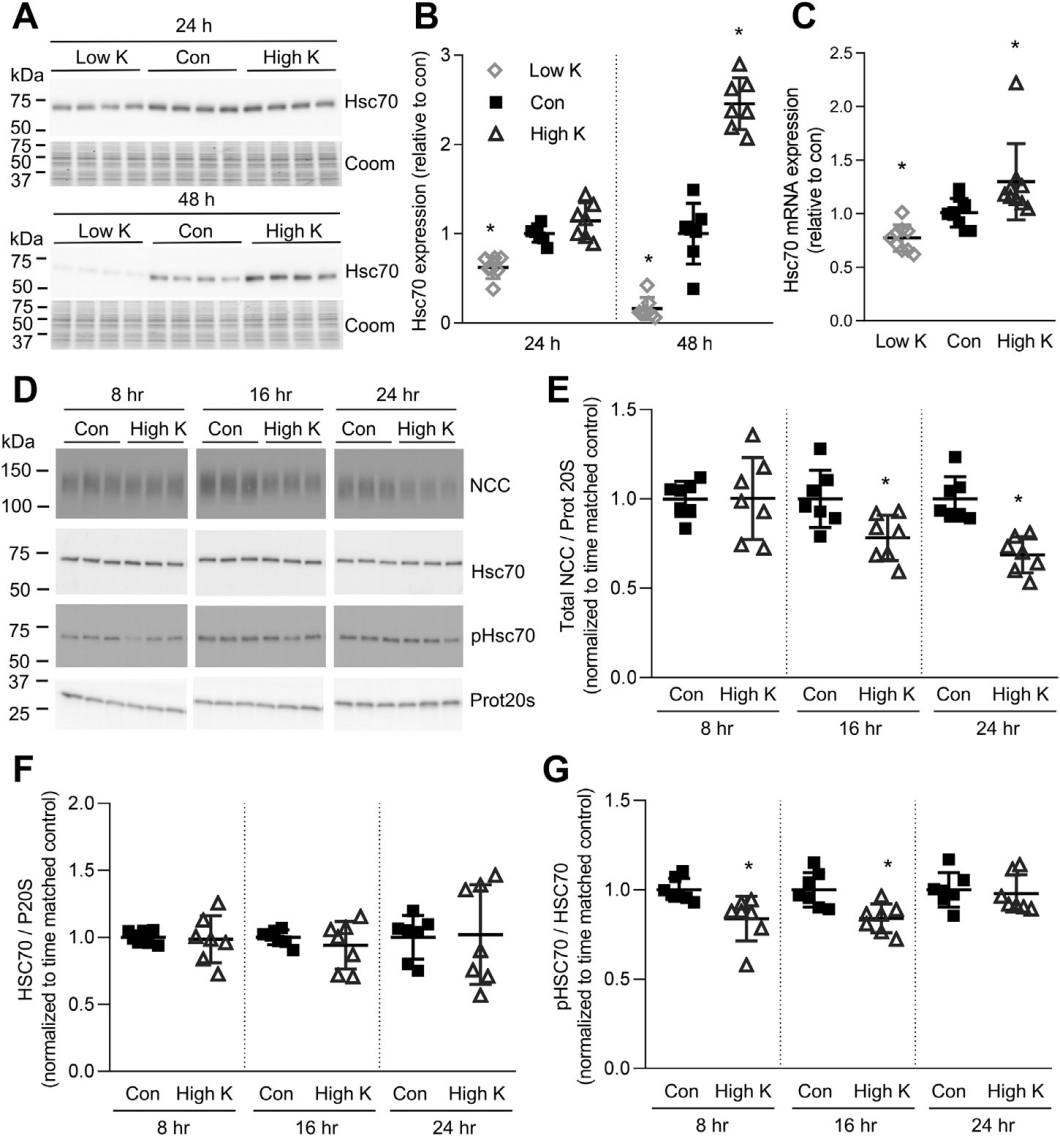
**Heat shock protein expression and phosphorylation are changed in response to high K**^**+**^. *A*, tubules were incubated in either low K^+^ (0.5 mM), control (con, 3.5 mM), or high K^+^ (8.0 mM) medium and Hsc70 levels assessed after 24 or 48 h. The same samples were used for blotting as for generating the data for Figure 1*A*, hence the Coomassie gels are the same. *B*, summary data (mean ± SD) of normalized band densities relative to control. ∗*p* < 0.05 compared with control media (n = 7). Comparisons were performed using a one-way ANOVA followed by a Dunnett multiple comparison test. *C*, similar studies assessing Hsc70 mRNA levels using RTqPCR. Signals are normalized against 18S rRNA and expressed relative to control (mean ± SD, n = 9). ∗*p* < 0.05 compared with control. Comparisons were performed using a one-way ANOVA followed by a Dunnett multiple comparison test. *D*, tubules were incubated in either control (Con, 3.5 mM K^+^) or high K^+^ (8.0 mM K^+^) medium for 8, 16, or 24 h. Tubules were harvested and subjected to immunoblotting for NCC, Hsc70, phosphorylated Hsc70, or Prot20s. Summary data for NCC (*E*), Hsc70 (*F*), and phosphorylated Hsc70/total Hsc70 (*G*) show normalized signal intensity relative to time matched control (n = 7) and significant differences are indicated (∗*p* < 0.05). *E–G*, shown are mean values ± SD. Comparisons were performed using an unpaired Student's *t* test. Coom, Coomassie blue staining.

Previous studies in cell lines have demonstrated that NCC forms complexes with the two cytoplasmic Hsp70s, Hsp70, and heat shock cognate 70 (Hsc70), as well as with CHIP and HOP, and that a CHIP-NCC interaction promotes NCC ubiquitylation and degradation (24, 25). In agreement, in lysates from primary tubule suspensions grown in control media NCC, Hsp70 and Hsc70 could be coimmunoprecipitated (Fig. 5*A*), suggesting they form a complex. To investigate if heat shock proteins are involved in K^+^-mediated NCC downregulation, tubules were incubated for 24 h with Ver-155008, a competitive inhibitor of Hsp70/Hsc70. In total, 150 μM Ver155008 significantly reduced Hsc70 expression, which correlated with significantly increased NCC expression (Fig. 5*B*). In the presence of Ver-155008, the ability of high K^+^ to reduce NCC abundance was also completely absent. After 48 h the effects of Ver-155008 were more pronounced (Fig. S4*B*). Taken together, this suggests that Hsp70/Hsc70 are involved in the normal biogenesis of NCC and play an essential role in mediating high K^+^-induced NCC degradation. Supporting this idea, in primary tubules incubated in different concentrations of K^+^ for 24–48 h, Hsc70 levels increased in parallel with increasing K^+^ levels (Fig. 6, *A* and *B*). These K^+^-induced changes in Hsc70 are at least in part dependent on altered transcription, as Hsc70 (*Hspa8*) mRNA expression increased with increasing K^+^ levels (Fig. 6*C*).

Whether interaction with Hsp70/Hsc70 results in ubiquitin-dependent degradation depends on their interaction with other proteins including CHIP and HOP. Dephosphorylated Hsp70/Hsc70 preferentially binds to CHIP, enhancing degradation of the client protein (23). In isolated tubules incubated in high K^+^ media for 8 and 16 h, phosphorylated Hsc70/Hsp70 was significantly decreased compared with tubules grown in control media (Fig. 6, *D*–*G*). A significant reduction in NCC abundance was only apparent after 16 h, suggesting that the reduction in phosphorylated Hsc70/Hsp70 precedes the high K^+^-mediated NCC degradation.

### A role for PP1α in mediating the long-term effects of high K^+^ on NCC abundance

Protein phosphatase 1 can bind and dephosphorylate members of the Hsp70 family (23, 26, 27). Theoretically this interaction could dephosphorylate Hsp70/Hsc70 leading to association of NCC with CHIP and subsequent targeting for degradation. PP1 activity has been linked to short-term modulation of NCC function *via* the protein phosphatase 1 inhibitor-1 (I-1) (28, 29), and the abundance of the alpha isoform of the PP1 catalytic subunit (PP1α) increases specifically in the DCT of mice fed a high K^+^ diet for 4 days (22). However, a role of PP1 for modulating NCC abundance has not been investigated.

**Figure 7:**
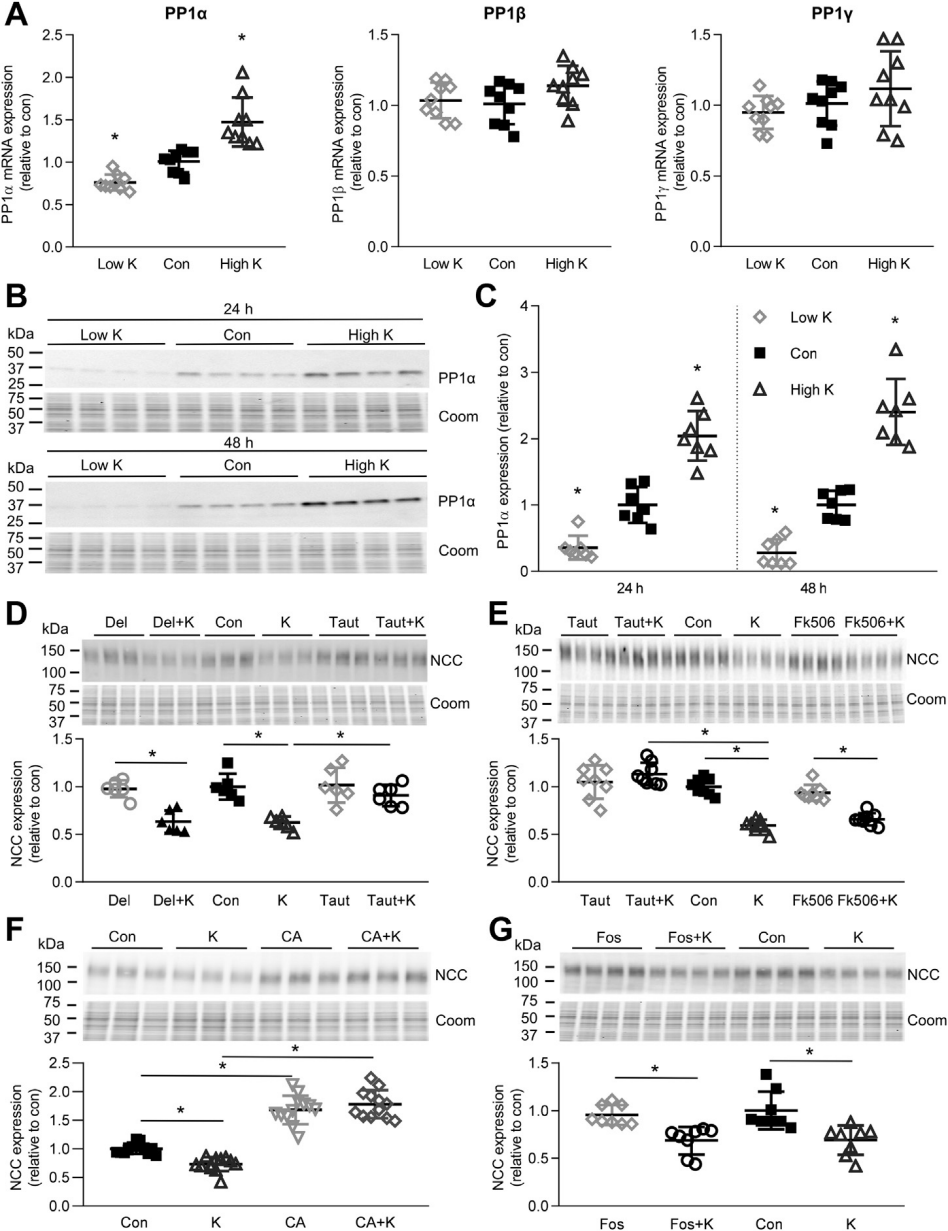
**Inhibition of PP1α prevents the ability of high K**^**+**^**to reduce total NCC levels in isolated renal cortical tubules.***A*, tubules were incubated in either low K^+^ (0.5 mM K^+^), control (Con, 3.5 mM K^+^), or high K^+^ (8.0 mM K^+^) medium for 24 h and PP1α, PP1β, or PP1γ mRNA levels were assessed using RTqPCR. Signals are normalized against 18S rRNA and expressed relative to control (mean ± SD, n = 9). ∗*p* < 0.05 compared with control media. Comparisons were performed using a one-way ANOVA followed by a Dunnett multiple comparison test. *B*, tubules were incubated in either low K^+^ (0.5 mM K^+^), control (Con, 3.5 mM K^+^), or high K^+^ (8.0 mM K^+^) medium and PP1α protein levels assessed after 24 or 48 h. The same samples were used for blotting as for generating the data for Figure 1*A*, hence the Coomassie gels are the same. *C*, summary data (mean ± SD) of normalized band densities relative to control (n = 7). ∗*p* < 0.05 compared with control media. Comparisons were performed using a one-way ANOVA followed by a Dunnett multiple comparison test. *D*, tubules were incubated in control (Con, 3.5 mM K^+^) or high K^+^ medium (K, 8.0 mM K^+^) for 24 h, with or without the PP1 inhibitor tautomycetin (Taut) or the PP2B/PP3 inhibitor deltamethrin (Del), and NCC levels were assessed by immunoblotting. Summary data show normalized band densities relative to control and significant differences are indicated (∗*p* < 0.05, n = 6). *E*, similar experiment using tautomycetin (Taut) or the PP2B/PP3 inhibitor Fk506 (n = 8). *F*, similar experiment using the PP1/PP2A inhibitor Calyculin A (CA) (n = 12). *G*, similar experiment using the PP2A inhibitor fostriecin (Fos) (n = 8). *D–G*, shown are mean values ± SD. Comparisons were performed using a two-way ANOVA followed by a Tukey multiple comparison test. Coom, Coomassie blue staining.

To investigate if K^+^ has a direct effect on PP1 expression, primary tubules were grown in different concentrations of K^+^. PP1α (*Ppp1ca*) mRNA expression increased after high K^+^ and decreased after low K^+^ (Fig. 7*A*), whereas alterations in K^+^ had no effect on mRNA levels of the PP1β (*Ppp1cb*) or PP1γ (*Ppp1cg*) subunits (Fig. 7*A*). In agreement with the changes in mRNA, PP1α protein levels were significantly lower after 24–48 h incubation in 0.5 mM K^+^ and increased after incubation in 8 mM K^+^ relative to control media (3.5 mM K^+^) (Fig. 7, *B* and *C*), a mirror opposite of what was observed with NCC (Fig. 1*A*). Experiments balancing altered KCl concentrations with NaCl (instead of choline Cl) showed similar results (Fig. S5*A*). Furthermore, aldosterone did not alter PP1α levels nor did it affect the high K^+^-mediated increase in PP1α (Fig. S5*B*).

In lysates from primary tubule suspensions grown in control media, NCC and PP1α could also be coimmunoprecipitated (Fig. 5*A*), suggesting they form a complex. The interaction of NCC with PP1α and Hsc70/Hsp70 was further investigated by growing tubules in high K^+^ or control medium and subsequently performing coimmunoprecipitations using an antibody for total NCC. In input samples, NCC expression decreased, as well as phosphorylated Hsc70/Hsp70, in agreement with a PP1-mediated dephosphorylation of Hsc70. The coimmunoprecipitated samples show that the interaction with NCC is not changed by incubating in high K^+^ medium, suggesting that this complex is present independent of K^+^ concentration (Fig. S6).

To confirm a role of PP1 activity for modulating the long-term effects of high K^+^ on NCC, tubules were incubated for 24 h in control or high K^+^ media with or without the PP1-inhibitor tautomycetin. The decrease in total NCC observed in high K^+^ medium was completely blocked by tautomycetin (Fig. 7, *D* and *E*), confirming that PP1 is involved in the long-term effects of K^+^ on NCC. In agreement with the effect of tautomycetin, the PP1/PP2A inhibitor Calyculin A increased total NCC expression and blocked the decrease in total NCC observed in high K^+^ medium (Fig. 7*F*). A role for PP2B/PP3 (calcineurin) in mediating the short-term effects of high K^+^ on phosphorylated NCC has been proposed (30). However, inhibiting PP2B/PP3 with deltamethrin (Fig. 7*D*) or Fk506 (tacrolimus) (Fig. 7*E*) did not prevent the decrease in NCC following 24 h of high K^+^. At high concentrations, tautomycetin and Calyculin A can inhibit PP2A (31). However, the PP2A/PP4 inhibitor fostriecin (32) did not affect total NCC levels relative to control or prevent the decrease in NCC with high K^+^ (Fig. 7*F*). Fostriecin increased phosphorylated Gsk3β, a known PP2A target (33), confirming inhibition of PP2A (Fig. S7). Together these data confirm that increased PP1 activity during long-term high K^+^ exposure plays a critical role in controlling NCC levels.

PP1 activity has been linked to short-term modulation of NCC function *via* the PP1 inhibitor I-1 (28, 29). Incubation of cortical tubules for 24 h in 0.5 mM K^+^ significantly decreased I-1 expression compared with control (3.5 mM), while high K^+^ had no effect. This effect of low K^+^ on I-1 was still apparent after 48 h, but at this time point I-1 levels were also significantly increased by high (8.0 mM) K^+^ (Fig. S8). The increased I-1 expression with high K^+^ after 48 h should decrease PP1 activity, which may counteract the increased PP1α expression.

### Effect of high K^+^ diet on PP1α and Hsc70 *in vivo*

**Figure 8:**
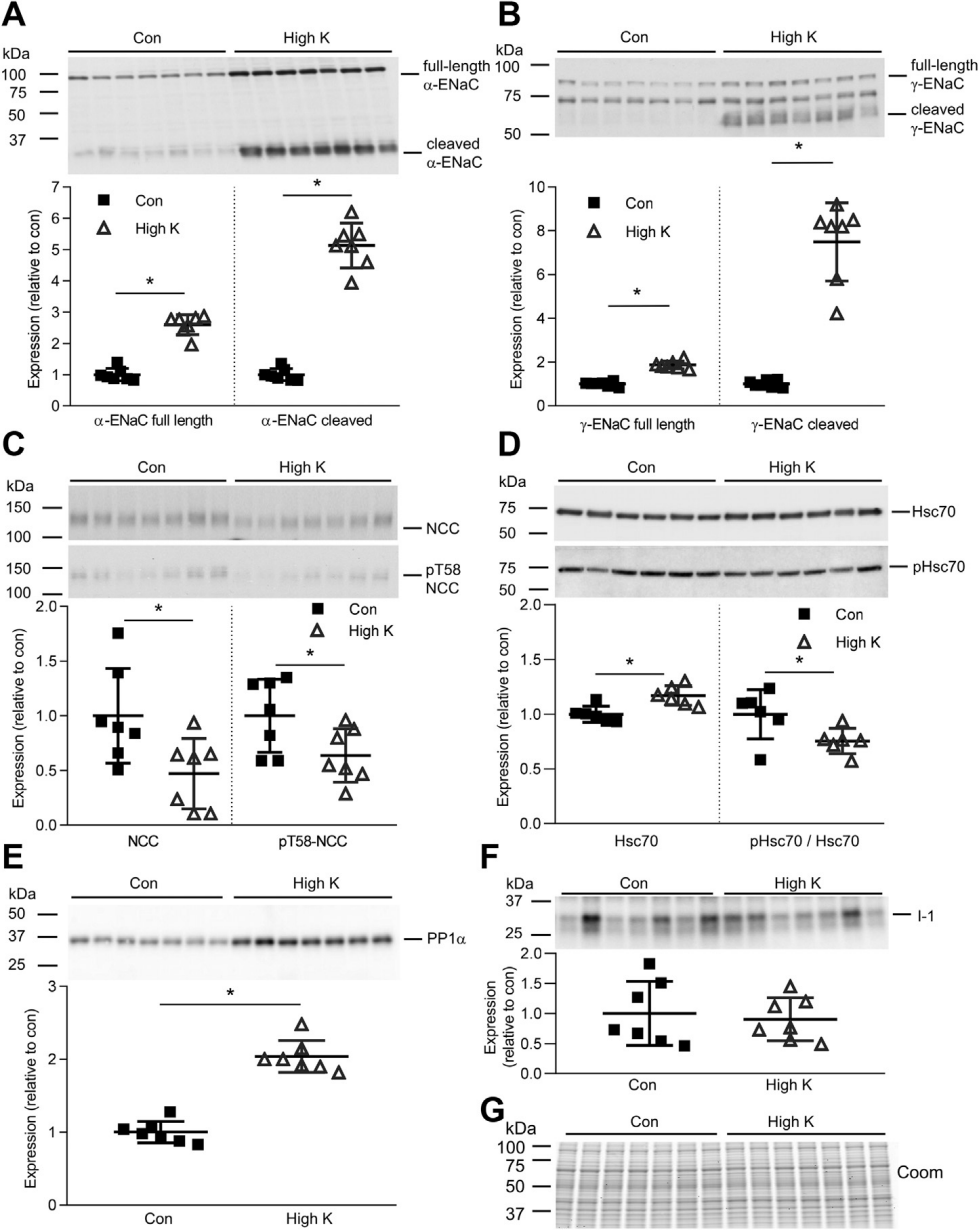
**Effect of high K**^**+**^***in vivo***. Immunoblotting of (*A*) α-ENaC (85 kDa full length and 30 kDa cleaved form), (*B*) γ-ENaC (95 kDa full length and 70 kDa cleaved form), (*C*) total NCC and phosphorylated T58 NCC (pT58 NCC), (*D*) Hsc70 and phosphorylated Hsc70, and (*E*) PP1α and (*F*) I-1 in cortical kidney homogenates of mice given a control (con) or high K^+^ diet for 4 days. Summary data of normalized band densities relative to control (n = 7 or 6) are shown (mean values ± SD). ∗*p* < 0.05 compared with control diet. Comparisons were performed using an unpaired Student's *t* test. *G*, Coomassie blue staining of the same samples used in blotting.

To investigate if findings in primary tubules could be confirmed *in vivo,* mice were fed a high K^+^ or control diet for 4 days. Altered physiological parameters of the mice under high dietary K^+^ intake included increased plasma aldosterone levels and greater urinary excretion of K^+^ (Table S1). In kidney cortex samples isolated from these mice, expression and cleavage of the α- and γ-ENaC subunits (34, 35) were significantly increased by increased dietary K^+^, whereas NCC and phosphorylated (active) T58-NCC levels were decreased (Fig. 8, *A*–*C*). PP1α levels were significantly increased in the renal cortex of the high K^+^-treated mice (Fig. 8*E*). In addition, Hsc70 levels were increased, while phosphorylated Hsc70/Hsp70 was decreased in the renal cortex of mice during high dietary K^+^ intake (Fig. 8*D*). No significant change was observed in the expression of I-1 (Fig. 8*F*). Together, these *in vivo* data support our findings in *ex vivo* primary tubules.

## Discussion

A high dietary K^+^ intake is associated with lower BP and a reduction in cardiovascular disease (6, 7, 8). The actions of NCC are thought to play a central role in the renal response to altered dietary K^+^ intake, with increased NCC abundance during low K^+^ intake, but lower NCC levels during high K^+^ intake (14, 15, 36). The mechanism of dietary K^+^-dependent NCC downregulation is unclear. Therefore, this study aimed to decipher the molecular mechanism responsible for reducing total NCC levels during high dietary K^+^ intake. A major finding is that high K^+^ does not alter NCC transcription, but increases ubiquitin-dependent degradation processes to reduce NCC abundance. Increased PP1 activity, likely acting *via* altering Hsp70-mediated degradation, is critical for this process.

A time-dependent increase in general protein ubiquitylation occurred in *ex vivo* tubule suspensions incubated in high K^+^. The mechanism responsible for this increase is unknown, but during the period of study it was not associated with altered cell integrity, cell death, decreased mitochondrial metabolic activity, or changes in proteins implicated in ER stress (not shown). NCC can also be ubiquitylated (19), which plays an important role in NCC degradation by the proteasome *via* endoplasmic-reticulum-associated degradation (ERAD) and by the lysosome (24, 37). Supporting a mechanism by which high K^+^ increases ubiquitin-dependent degradation of NCC, high K^+^ exposure increased NCC ubiquitylation during inhibition of the lysosomal or the proteasomal pathways, but NCC levels were not decreased. The importance of ubiquitin-mediated NCC degradation was further emphasized by the absence of high K^+^ effects on NCC when the ubiquitin-activating enzyme E1 was inhibited.

**Figure 9:**
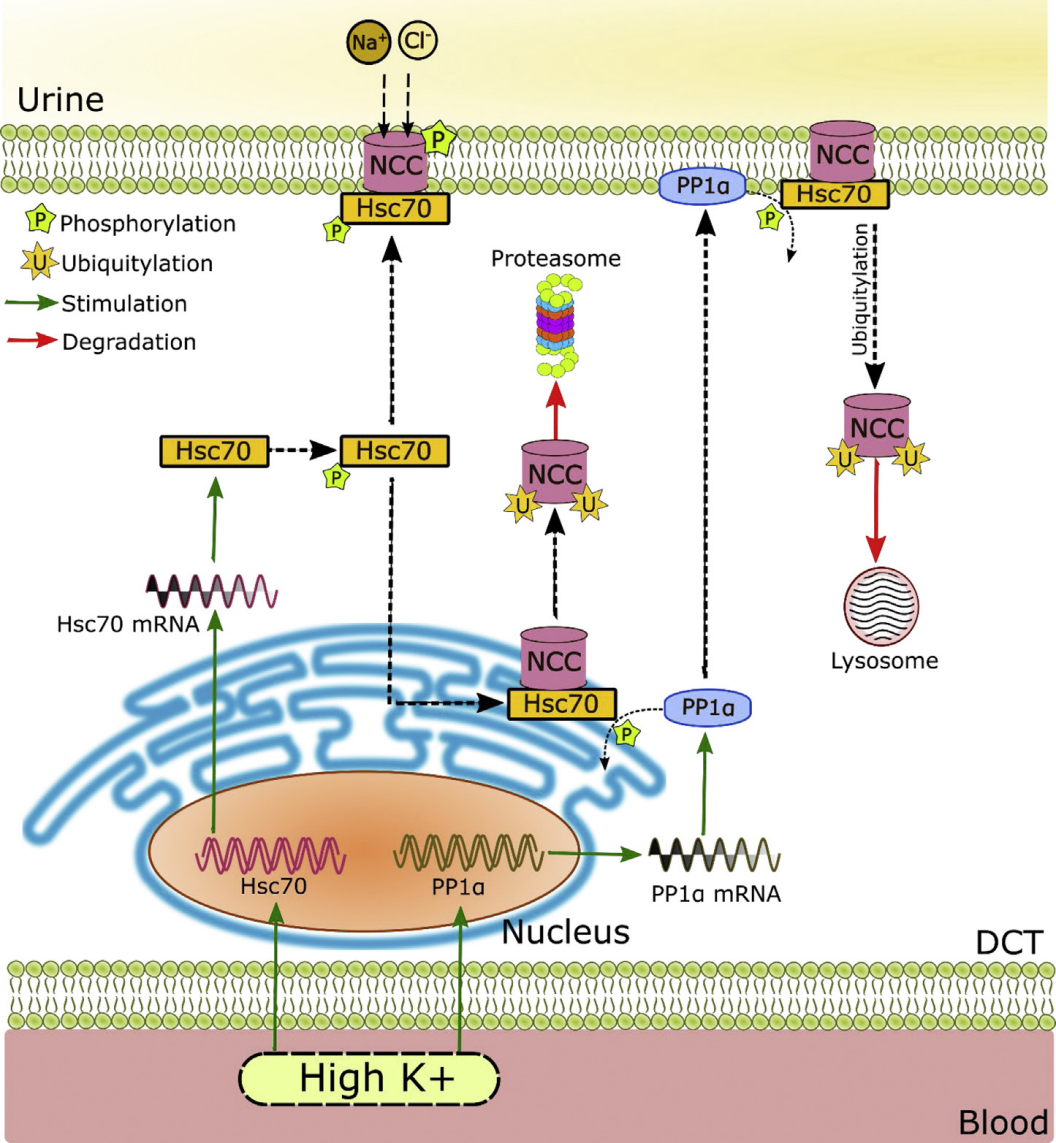
**Schematic of the role of PP1α and Hsc70 in hyperkalemia-driven NCC degradation**. Hyperkalemia increases mRNA and protein expression of both Hsc70 and PP1α. The chaperone protein Hsc70 plays an essential role in both protein folding and ubiquitin-dependent protein degradation, depending on its phosphorylation. Hyperkalemia increases PP1α-mediated dephosphorylation of Hsc70, promoting ubiquitylation and degradation of NCC. As part of the ER quality control, Hsc70-bound NCC can be targeted to endoplasmic-reticulum-associated degradation (ERAD) *via* the ubiquitin proteasome system. Hsc70 is also involved in peripheral quality control at the plasma membrane and could target NCC to lysosomal degradation.

What is the mechanism for the high K^+^-induced ubiquitylation and degradation of NCC? Although this is likely to be multifactoral, we propose that the major pathway relates to the effects of increased activity of PP1α on the function of heat shock protein 70 (Hsp70) (see Fig. 9). The chaperone protein Hsp70 plays an essential role in both protein folding and ubiquitin-dependent protein degradation (21). Dephosphorylation of the two cytoplasmic Hsp70s, the constitutively expressed Hsc70 and stress-induced Hsp70, by PP1 enhances the interaction of Hsp70-bound proteins with the cochaperone CHIP, promoting ubiquitylation and degradation of client proteins (23, 26, 27). This process is important both for proteasomal degradation of misfolded proteins *via* ERAD and for peripheral quality control where damaged proteins are endocytosed and targeted for lysosomal degradation (38). Phosphorylated Hsc70/Hsp70 levels were decreased in the renal cortex of mice given a high K^+^ diet, while PP1α and Hsc70 levels were increased. Similar alterations in PP1α, Hsc70, and phosphorylated Hsc70/Hsp70 levels could be mimicked in cortical tubules by increasing K^+^ levels. These changes would together facilitate a shift in balance within the DCT toward degradation of Hsc70 bound proteins. Supporting this mechanism NCC, PP1α, and Hsc70 were found to form a complex *ex vivo*, and previous studies have demonstrated that a NCC-CHIP interaction promotes NCC ubiquitylation and degradation (24, 25). Furthermore, in tubule studies the decrease in phosphorylated Hsc70/Hsp70 levels preceded the reduction in total NCC levels, suggesting that this is a trigger for effects on NCC. Finally, and perhaps most critically, functional inhibition of either PP1 or Hsc70 activity prevented the high K^+^-induced degradation of NCC. How high K^+^ increases PP1α or Hsc70, or whether activation of Hsc70 *in vivo* can mimic the effects of high K^+^ is unknown. The role of CHIP and another E3 ubiquitin ligase Nedd4-2 (39) in the response to high K^+^ is the focus of ongoing studies, but it has been recently demonstrated that dietary Mg^2^ restriction decreases NCC abundance *via* Nedd4-2 and that this overrides the stimulatory effect of dietary K^+^ restriction on NCC (40).

An unanswered question is how Kir4.1/Kir5.1 fits into the mechanism? Raising extracellular K^+^ transiently increases the intracellular Cl^−^ concentration in the DCT, and blockade of Kir4.1/Kir5.1 or inhibition of the Cl^−^ channel ClC-K2 increases intracellular Cl^−^ concentrations (41). Hence, diet-induced changes in the extracellular K^+^ concentration are thought to alter the basolateral plasma membrane potential *via* Kir4.1/Kir5.1, resulting in altered intracellular Cl^−^ levels and ultimately modulation of the Cl^−^ sensitive lysine deficient protein kinase (WNK) 1 and 4, affecting the WNK-SPAK/OSR1 kinase signaling pathway and NCC phosphorylation (10, 11). In K^+^ deficiency, WNKs and SPAK/OSR1 concentrate in spherical cytoplasmic domains in the DCT termed "WNK bodies," in which WNK4 is the primary active WNK isoform catalyzing SPAK/OSR1 phosphorylation. These WNK bodies do not develop in cells lacking Kir4.1 (42). The ability of high K^+^ to reduce NCC abundance is also reduced in mice lacking Kir4.1/Kir5.1 (12, 13). As there is an inverse correlation between NCC phosphorylation and NCC ubiquitylation (43, 44), it could be speculated that total NCC levels do not decrease in response to high K^+^ in mice lacking Kir4.1/Kir5.1 simply because NCC phosphorylation is not altered. Alternatively, it could be that the activity of Kir4.1/Kir5.1 is central to driving the PP1α/Hsc70/ubiquitin-dependent NCC degradation mechanism uncovered here.

In mice, high dietary K^+^ strongly increased full length and cleaved α and γ-ENaC abundance, which in part is likely a result of increased release of aldosterone from the adrenal gland subsequent to increased plasma K^+^ (34, 35). In addition, K^+^ can stimulate ENaC independently of aldosterone in mpkCCD cells (17). In line with this, in this study low K^+^ directly decreased α-ENaC abundance in cortical tubules, although high K^+^ had no significant effect. Hsp70 has also been shown to promote ENaC functional expression by increasing its association with coat complex II and its exit from the endoplasmic reticulum (45). K^+^-induced changes in Hsp70 could therefore also play a role in the effect of K^+^ on ENaC. However, Hsc70 and Hsp70 have differential and antagonistic effects with regard to the intracellular trafficking of ENaC in oocytes, with Hsc70 decreasing but Hsp70 increasing the functional and surface expression of ENaC (46). Whether Hsc70 or Hsp70 plays a role in ENaC regulation requires further studies.

In conclusion, we provide a novel mechanism for NCC reduction following a high K^+^ diet. Increased protein phosphatase 1α (PP1α) activity subsequent to high K^+^ intake drives ubiquitin-dependent NCC degradation in a mechanism facilitated by heat shock protein 70. This mechanism helps increase K^+^ secretion during hyperkalemia and is a novel concept for understanding how high dietary K^+^ can reduce BP.

## Experimental procedures

### Antibodies

Specificity of the commercial antibodies was based on that they either gave a single unique band on an immunoblot corresponding to the target proteins predicted molecular weight, or the most prominent band on the immunoblot was at the target proteins predicted molecular weight (with no other bands of similar size). The following antibodies were used: a rabbit polyclonal NCC antibody (SPC-402D, StressMarq), phosphorylated Threonine-58 NCC (pT58) antibody (47), mouse antiubiquitin antibody (P4D1, cell signaling), mouse PPP1ca antibody (Thermofisher), rabbit Phospho-GSK-3β (Ser9) antibody (cell signaling) rat HSC70 antibody (Enzo), mouse HSP70 antibody (Enzo), mouse monoclonal antibody clone GGS2.1 recognizing the phosphorylated C-terminal threonine of Hsp70/Hsc70 GSGP(pT)IEEVD(23), rabbit I-1 antibody (ab40877 Abcam), rabbit α-ENaC antibody (48), and rabbit γ-ENaC antibody (34). An additional mouse monoclonal anti-NCC (SLC12A3) antibody was developed by immunization of mice with the NCC epitope GEPRKVRPTLADLHSFLKQEG as previously detailed (49). From various clones, clone 3 was suitable for immunoblotting and used in this study. Detection of NCC in mouse kidney lysates was validated by western blotting on tissue obtained from wild-type and NCC-deficient mice (50) (generously provided by Régine Chambrey, INSERM) (Fig. S9*A*). In addition, HEK293 cells were transfected with human *SLC12A3* (51) or mock using METAFECTENE (Biontex) according to manufacturer's instructions, and NCC expression was induced using doxycycline. Lysates were analyzed using the monoclonal antibody by western blotting (Fig. S9*B*).

### Animal experiments

All protocols were approved and performed under a license issued for the use of experimental animals by the Danish Ministry of Justice (Dyreforsøgstilsynet). C57Bl/6J mice were kept in standard cages in a room with a 12:12-h artificial light–dark cycle, a temperature of 21 ± 2 °C, and humidity of 55 ± 2%, with free access to tap water and standard rodent diet (1324 pellets, Altromin). For experiments, mice were housed individually in metabolic cages, in a room at 27 °C, and during a 2-day acclimatization period received a control diet (0.32% Na^+^, 0.38% K^+^). Mice were switched to either a high K^+^ (5% K^+^) diet or control diet (0.38% K^+^, containing 0.32% Na^+^, Zeigler Brothers, Gardners, PA, USA) for 4 days. The control diet was prepared by adding NaCl (8.13 g/kg food) dissolved in 300 ml water/kg food to a low NaCl diet to obtain a final concentration of 0.32% Na^+^. The K^+^ diet was prepared by adding NaCl (8.13 g/kg food) and 127.8 g/kg potassium citrate dissolved in 300 ml/kg food to obtain final concentrations of 0.32% Na^+^ and 5% K^+^. Water intake was calculated by adding the water in the food to the drinking volume.

### Osmolality and electrolyte measurements

Collected urine was centrifuged at 1000 *g* for 1 min to clear sediments. Osmolality was measured on a Micro Osmometer 3320 (Advanced Instruments Inc). Blood plasma was prepared by collecting blood from the retro-orbital plexus in Li-heparin tubes, inverting 8–10 times, followed by centrifugation. Urine and plasma sodium, chloride, potassium, urea, and creatinine concentrations were analyzed by the Clinical Pathology Laboratory at the Medical Research Council (Harwell). Plasma aldosterone concentrations were determined using an enzyme immunoassay kit (EIA-5298; range: 20–1000 pg/ml; QC: standards, DRG International).

### Immunoblotting

Kidneys were isolated and the cortex was dissected. Tissue was homogenized in ice-cold dissection buffer (0.3 M sucrose, 25 mM imidazole, and 1 mM EDTA, pH 7.2) containing Complete protease inhibitor tablets and PhosSTOP phosphatase inhibitor tablets (both from Roche Diagnostics) followed by a low-velocity spin (1000 *g*, 5 min, 4 °C). Standard procedures were utilized for sample preparation and SDS-PAGE using 4–15% gradient polyacrylamide gels (Criterion TGX Precast Protein Gels, BioRad). Equal quantities of total protein were loaded per lane as determined by Coomassie blue staining. The maximal deviations in total protein concentration between samples on individual blots were ±10%. Immunoblots were developed using SuperSignal West Femto chemiluminescent substrate (Thermo Scientific) or Amersham ECL Western Blotting Detection Reagent (GE Healthcare). Signal intensity in specific bands was quantified using Image Studio Lite (Qiagen) densitometry analysis.

### *Ex vivo* renal cortical tubules

Male C57/bl6/J mice were killed by cervical dislocation, and kidneys were removed. The cortex was dissected into ∼1-mm pieces and placed into 4 ml of enzyme solution containing 1.5 mg/ml collagenase type B (Roche) in buffer B (125 mM NaCl, 30 mM glucose, 0.4 mM KH_2_PO_4_ 1.6 mM K_2_HPO_4_, 1 mM MgSO_4_, 10 mM Na-acetate, 1 mM α-ketogluterate, 1.3 mM Ca-gluconate, 5 mM glycine, 48 μg/ml trypsin inhibitor, and 50 μg/ml DNase, pH 7.4). Samples were mixed continuously at 37 °C and 850 rpm. After 10 min, 2 ml of fresh enzyme solution was added. After an additional 10 min, half of the enzyme solution was placed on ice and replaced with 2 ml of buffer B, which was repeated after an additional 10 min, during which the remaining fragments were pipetted up and down ten times. After a total period of 40 min, all tubules were centrifuged for 2 min at 200*g*. The pellet was resuspended in 5 ml of potassium-free DMEM (110 mM NaCl, 26 mM NaHCO_3_, 0.81 mM MgSO_4_, 1.8 mM CaCl_2_, 2.48 μM Fe(NO_3_)_3_, 0.91 mM NaH_2_PO_4_, 25 mM glucose, 1 mM sodium pyruvate, 0.4 mM glycine, 4 mM L-glutamine), MEM vitamin solution (Thermo Fisher), and MEM amino acids (Thermo Fisher), mixed *via* pipetting, and centrifuged at 200*g* for 2 min. The tubular suspensions were resuspended in DMEM and transferred into 24-well plates. Tubules were incubated at 37 °C and 5% CO_2_. Potassium concentrations were changed by adding KCl or potassium citrate as indicated. A similar concentration of choline Cl was added to control media. Tubule viability through the duration of the experiments was determined using (1) brightfield microscopy, (2) cell counting and propidium iodine staining using a NucleoCounter NC-100 cytometer, (3) the redox-sensitive dye resazurin (R + D Systems). Concentrations of chemicals used are 100 μM chloroquine, 10 μM Mg132, 25 or 150 μM Ver-15508, 10 nM deltamethrin, 10 nM aldosterone (all Sigma), 50 or 150 μM Pyr-41, 160 nM tautomycetin, 10 μM FK506, 1.5 μM fostriecin, or 10 nM Calyculin-A (all Tocris). Tubules were harvested in Laemmli sample buffer containing 100 mM DTT, sonicated, and heated for 30 min at 37 °C.

### Immunoprecipitation

Tubules were incubated as described above and lysed in lysis buffer (20 mM Tris, 135 mM NaCl, 1% Triton, 5 mM EDTA [pH 7.4]) containing 20 mM N-ethylmaleimide (Sigma), 22 μM PR619 (Abcam), Complete protease inhibitor tablets and PhosSTOP phosphatase inhibitor tablets (both from Roche Diagnostics) for 30 min at 4 °C to solubilize membrane proteins. Samples were assayed for protein concentration and subjected to immunoprecipitation using 5 μg of rabbit NCC antibody or 1 μg of PP1α antibody and 20 μl of protein A-agarose or G plus-agarose (Santa Cruz Biotechnology) at 4 °C overnight with rotation followed by washing three times with lysis buffer and elution in laemmli buffer with 100 mM DTT. To isolate ubiquitinated proteins, a high-binding affinity matrix based on a ubiquitin antibody tagged to agarose beads (UBI-QAPTURE-Q matrix, Enzo Life Scienes) was used according to manufacturer's instructions.

### Real time quantitative PCR (RTqPCR)

Renal cortical tubules were isolated and cultured as described above. Total RNA was isolated using Trizol reagent and a Ribopure RNA purification kit (Invitrogen), according to the manufacturer's instructions. To remove genomic DNA, total RNA was treated with DNase I, Amplification Grade (Invitrogen), and RNA was reverse-transcribed into cDNA using random primers and Superscript II Reverse Transcriptase (Invitrogen). During cDNA production, a control reaction without the reverse transcriptase enzyme was included to exclude genomic DNA amplification. Exon overlapping primers were used, see Table S2. RTqPCR was performed using SYBR Green I Master Mix (Roche) on a lightcycler 480 (Roche). Signals for ribosomal 18S were used to normalize for differences in the amount of starting cDNA (sequence in Table S2).

### Statistical analysis

For western blotting and mouse physiological measurements, data are expressed as mean ± standard deviation (SD). For two groups, data meeting the statistical assumptions of normality were assessed using an unpaired Student's *t* test. Comparisons of more than two groups were performed using a one-way or two-way ANOVA followed by a Tukey or Dunnett multiple comparison test. Sample number (n) is highlighted in figure legends. Significance was considered at *p* < 0.05.

## Data availability

All data are contained within the manuscript or supplemental materials.

## Supporting information

This article contains supporting information.

## Conflicts of interest

The authors declare that they have no conflicts of interest with the contents of this article.

## References

[1] Kearney P.M., Whelton M., Reynolds K., Whelton P.K., He J. (2004). Worldwide prevalence of hypertension: A systematic review. J. Hypertens..

[2] Guyton A.C. (1991). Blood pressure control--special role of the kidneys and body fluids. Science.

[3] Simon D.B., Nelson-Williams C., Bia M.J., Ellison D., Karet F.E., Molina A.M., Vaara I., Iwata F., Cushner H.M., Koolen M., Gainza F.J., Gitleman H.J., Lifton R.P. (1996). Gitelman's variant of Bartter's syndrome, inherited hypokalaemic alkalosis, is caused by mutations in the thiazide-sensitive Na-Cl cotransporter. Nat. Genet..

[4] Wilson F.H., Disse-Nicodeme S., Choate K.A., Ishikawa K., Nelson-Williams C., Desitter I., Gunel M., Milford D.V., Lipkin G.W., Achard J.M., Feely M.P., Dussol B., Berland Y., Unwin R.J., Mayan H. (2001). Human hypertension caused by mutations in WNK kinases. Science.

[5] Yang C.L., Angell J., Mitchell R., Ellison D.H. (2003). WNK kinases regulate thiazide-sensitive Na-Cl cotransport. J. Clin. Invest..

[6] Mente A., O'Donnell M.J., Rangarajan S., McQueen M.J., Poirier P., Wielgosz A., Morrison H., Li W., Wang X., Di C., Mony P., Devanath A., Rosengren A., Oguz A., Zatonska K. (2014). Association of urinary sodium and potassium excretion with blood pressure. N. Engl. J. Med..

[7] O'Donnell M., Mente A., Rangarajan S., McQueen M.J., Wang X., Liu L., Yan H., Lee S.F., Mony P., Devanath A., Rosengren A., Lopez-Jaramillo P., Diaz R., Avezum A., Lanas F. (2014). Urinary sodium and potassium excretion, mortality, and cardiovascular events. N. Engl. J. Med..

[8] Akita S., Sacks F.M., Svetkey L.P., Conlin P.R., Kimura G., DASH-Sodium Trial Collaborative Research Group (2003). Effects of the dietary approaches to stop hypertension (DASH) diet on the pressure-natriuresis relationship. Hypertension.

[9] Terker A.S., Zhang C., McCormick J.A., Lazelle R.A., Zhang C., Meermeier N.P., Siler D.A., Park H.J., Fu Y., Cohen D.M., Weinstein A.M., Wang W.H., Yang C.L., Ellison D.H. (2015). Potassium modulates electrolyte balance and blood pressure through effects on distal cell voltage and chloride. Cell Metab..

[10] Piala A.T., Moon T.M., Akella R., He H., Cobb M.H., Goldsmith E.J. (2014). Chloride sensing by WNK1 involves inhibition of autophosphorylation. Sci. Signal.

[11] Bazua-Valenti S., Chavez-Canales M., Rojas-Vega L., Gonzalez-Rodriguez X., Vazquez N., Rodriguez-Gama A., Argaiz E.R., Melo Z., Plata C., Ellison D.H., Garcia-Valdes J., Hadchouel J., Gamba G. (2015). The effect of WNK4 on the Na+-Cl- cotransporter is modulated by intracellular chloride. J. Am. Soc. Nephrol..

[12] Cuevas C.A., Su X.T., Wang M.X., Terker A.S., Lin D.H., McCormick J.A., Yang C.L., Ellison D.H., Wang W.H. (2017). Potassium sensing by renal distal tubules requires Kir4.1. J. Am. Soc. Nephrol..

[13] Wu P., Gao Z.X., Zhang D.D., Su X.T., Wang W.H., Lin D.H. (2019). Deletion of Kir5.1 impairs renal ability to excrete potassium during increased dietary potassium intake. J. Am. Soc. Nephrol..

[14] Vallon V., Schroth J., Lang F., Kuhl D., Uchida S. (2009). Expression and phosphorylation of the Na+-Cl- cotransporter NCC *in vivo* is regulated by dietary salt, potassium, and SGK1. Am. J. Physiol. Renal Physiol..

[15] Frindt G., Palmer L.G. (2010). Effects of dietary K on cell-surface expression of renal ion channels and transporters. Am. J. Physiol. Renal Physiol..

[16] Kim G.H., Masilamani S., Turner R., Mitchell C., Wade J.B., Knepper M.A. (1998). The thiazide-sensitive Na-Cl cotransporter is an aldosterone-induced protein. Proc. Natl. Acad. Sci. U. S. A..

[17] Sorensen M.V., Saha B., Jensen I.S., Wu P., Ayasse N., Gleason C.E., Svendsen S.L., Wang W.H., Pearce D. (2019). Potassium acts through mTOR to regulate its own secretion. JCI Insight.

[18] Melikova M.S., Kondratov K.A., Kornilova E.S. (2006). Two different stages of epidermal growth factor (EGF) receptor endocytosis are sensitive to free ubiquitin depletion produced by proteasome inhibitor MG132. Cell Biol. Int..

[19] Rosenbaek L.L., Rizzo F., Wu Q., Rojas-Vega L., Gamba G., MacAulay N., Staub O., Fenton R.A. (2017). The thiazide sensitive sodium chloride co-transporter NCC is modulated by site-specific ubiquitylation. Sci. Rep..

[20] Yang Y., Kitagaki J., Dai R.M., Tsai Y.C., Lorick K.L., Ludwig R.L., Pierre S.A., Jensen J.P., Davydov I.V., Oberoi P., Li C.C., Kenten J.H., Beutler J.A., Vousden K.H., Weissman A.M. (2007). Inhibitors of ubiquitin-activating enzyme (E1), a new class of potential cancer therapeutics. Cancer Res..

[21] Edkins A.L. (2015). CHIP: A co-chaperone for degradation by the proteasome. Subcell Biochem..

[22] Kortenoeven M.L.A., Cheng L., Wu Q., Fenton R.A. (2021). An *in vivo* protein landscape of the mouse DCT during high dietary K(+) or low dietary Na(+) intake. Am. J. Physiol. Renal Physiol..

[23] Muller P., Ruckova E., Halada P., Coates P.J., Hrstka R., Lane D.P., Vojtesek B. (2013). C-terminal phosphorylation of Hsp70 and Hsp90 regulates alternate binding to co-chaperones CHIP and HOP to determine cellular protein folding/degradation balances. Oncogene.

[24] Donnelly B.F., Needham P.G., Snyder A.C., Roy A., Khadem S., Brodsky J.L., Subramanya A.R. (2013). Hsp70 and Hsp90 multichaperone complexes sequentially regulate thiazide-sensitive cotransporter endoplasmic reticulum-associated degradation and biogenesis. J. Biol. Chem..

[25] Needham P.G., Mikoluk K., Dhakarwal P., Khadem S., Snyder A.C., Subramanya A.R., Brodsky J.L. (2011). The thiazide-sensitive NaCl cotransporter is targeted for chaperone-dependent endoplasmic reticulum-associated degradation. J. Biol. Chem..

[26] Polanowska-Grabowska R., Simon C.G., Falchetto R., Shabanowitz J., Hunt D.F., Gear A.R. (1997). Platelet adhesion to collagen under flow causes dissociation of a phosphoprotein complex of heat-shock proteins and protein phosphatase 1. Blood.

[27] Flores-Delgado G., Liu C.W., Sposto R., Berndt N. (2007). A limited screen for protein interactions reveals new roles for protein phosphatase 1 in cell cycle control and apoptosis. J. Proteome Res..

[28] Picard N., Trompf K., Yang C.L., Miller R.L., Carrel M., Loffing-Cueni D., Fenton R.A., Ellison D.H., Loffing J. (2014). Protein phosphatase 1 inhibitor-1 deficiency reduces phosphorylation of renal NaCl cotransporter and causes arterial hypotension. J. Am. Soc. Nephrol..

[29] Penton D., Moser S., Wengi A., Czogalla J., Rosenbaek L.L., Rigendinger F., Faresse N., Martins J.R., Fenton R.A., Loffing-Cueni D., Loffing J. (2019). Protein phosphatase 1 inhibitor-1 mediates the cAMP-dependent stimulation of the renal NaCl cotransporter. J. Am. Soc. Nephrol..

[30] Shoda W., Nomura N., Ando F., Mori Y., Mori T., Sohara E., Rai T., Uchida S. (2017). Calcineurin inhibitors block sodium-chloride cotransporter dephosphorylation in response to high potassium intake. Kidney Int..

[31] Mitsuhashi S., Matsuura N., Ubukata M., Oikawa H., Shima H., Kikuchi K. (2001). Tautomycetin is a novel and specific inhibitor of serine/threonine protein phosphatase type 1, PP1. Biochem. Biophys. Res. Commun..

[32] Walsh A.H., Cheng A., Honkanen R.E. (1997). Fostriecin, an antitumor antibiotic with inhibitory activity against serine/threonine protein phosphatases types 1 (PP1) and 2A (PP2A), is highly selective for PP2A. FEBS Lett..

[33] Marion S., Urs N.M., Peterson S.M., Sotnikova T.D., Beaulieu J.M., Gainetdinov R.R., Caron M.G. (2014). Dopamine D2 receptor relies upon PPM/PP2C protein phosphatases to dephosphorylate huntingtin protein. J. Biol. Chem..

[34] Masilamani S., Kim G.H., Mitchell C., Wade J.B., Knepper M.A. (1999). Aldosterone-mediated regulation of ENaC alpha, beta, and gamma subunit proteins in rat kidney. J. Clin. Invest..

[35] Ergonul Z., Frindt G., Palmer L.G. (2006). Regulation of maturation and processing of ENaC subunits in the rat kidney. Am. J. Physiol. Renal Physiol..

[36] Hoorn E.J., Gritter M., Cuevas C.A., Fenton R.A. (2020). Regulation of the renal NaCl cotransporter and its role in potassium homeostasis. Physiol. Rev..

[37] Subramanya A.R., Liu J., Ellison D.H., Wade J.B., Welling P.A. (2009). WNK4 diverts the thiazide-sensitive NaCl cotransporter to the lysosome and stimulates AP-3 interaction. J. Biol. Chem..

[38] Okiyoneda T., Apaja P.M., Lukacs G.L. (2011). Protein quality control at the plasma membrane. Curr. Opin. Cell Biol..

[39] Arroyo J.P., Lagnaz D., Ronzaud C., Vazquez N., Ko B.S., Moddes L., Ruffieux-Daidie D., Hausel P., Koesters R., Yang B., Stokes J.B., Hoover R.S., Gamba G., Staub O. (2011). Nedd4-2 modulates renal Na+-Cl- cotransporter via the aldosterone-SGK1-Nedd4-2 pathway. J. Am. Soc. Nephrol..

[40] Ferdaus M.Z., Mukherjee A., Nelson J.W., Blatt P.J., Miller L.N., Terker A.S., Staub O., Lin D.H., McCormick J.A. (2019). Mg(2+) restriction downregulates NCC through NEDD4-2 and prevents its activation by hypokalemia. Am. J. Physiol. Renal Physiol..

[41] Su X.T., Klett N.J., Sharma A., Allen C.N., Wang W.H., Yang C.L., Ellison D.H. (2020). Distal convoluted tubule Cl(-) concentration is modulated via K(+) channels and transporters. Am. J. Physiol. Renal Physiol..

[42] Thomson M.N., Cuevas C.A., Bewarder T.M., Dittmayer C., Miller L.N., Si J., Cornelius R.J., Su X.T., Yang C.L., McCormick J.A., Hadchouel J., Ellison D.H., Bachmann S., Mutig K. (2020). WNK bodies cluster WNK4 and SPAK/OSR1 to promote NCC activation in hypokalemia. Am. J. Physiol. Renal Physiol..

[43] Rosenbaek L.L., Kortenoeven M.L., Aroankins T.S., Fenton R.A. (2014). Phosphorylation decreases ubiquitylation of the thiazide-sensitive cotransporter NCC and subsequent clathrin-mediated endocytosis. J. Biol. Chem..

[44] Hossain Khan M.Z., Sohara E., Ohta A., Chiga M., Inoue Y., Isobe K., Wakabayashi M., Oi K., Rai T., Sasaki S., Uchida S. (2012). Phosphorylation of Na-Cl cotransporter by OSR1 and SPAK kinases regulates its ubiquitination. Biochem. Biophys. Res. Commun..

[45] Chanoux R.A., Robay A., Shubin C.B., Kebler C., Suaud L., Rubenstein R.C. (2012). Hsp70 promotes epithelial sodium channel functional expression by increasing its association with coat complex II and its exit from endoplasmic reticulum. J. Biol. Chem..

[46] Goldfarb S.B., Kashlan O.B., Watkins J.N., Suaud L., Yan W., Kleyman T.R., Rubenstein R.C. (2006). Differential effects of Hsc70 and Hsp70 on the intracellular trafficking and functional expression of epithelial sodium channels. Proc. Natl. Acad. Sci. U. S. A..

[47] Pedersen N.B., Hofmeister M.V., Rosenbaek L.L., Nielsen J., Fenton R.A. (2010). Vasopressin induces phosphorylation of the thiazide-sensitive sodium chloride cotransporter in the distal convoluted tubule. Kidney Int..

[48] Sorensen M.V., Grossmann S., Roesinger M., Gresko N., Todkar A.P., Barmettler G., Ziegler U., Odermatt A., Loffing-Cueni D., Loffing J. (2013). Rapid dephosphorylation of the renal sodium chloride cotransporter in response to oral potassium intake in mice. Kidney Int..

[49] Frische S., Chambrey R., Trepiccione F., Zamani R., Marcussen N., Alexander R.T., Skjodt K., Svenningsen P., Dimke H. (2018). H(+)-ATPase B1 subunit localizes to thick ascending limb and distal convoluted tubule of rodent and human kidney. Am. J. Physiol. Renal Physiol..

[50] Schultheis P.J., Lorenz J.N., Meneton P., Nieman M.L., Riddle T.M., Flagella M., Duffy J.J., Doetschman T., Miller M.L., Shull G.E. (1998). Phenotype resembling Gitelman's syndrome in mice lacking the apical Na+-Cl- cotransporter of the distal convoluted tubule. J. Biol. Chem..

[51] Rosenbaek L.L., Rizzo F., MacAulay N., Staub O., Fenton R.A. (2017). Functional assessment of sodium chloride cotransporter NCC mutants in polarized mammalian epithelial cells. Am. J. Physiol. Renal Physiol..

