# Supplementary material for: High dietary potassium causes ubiquitin-dependent degradation of the kidney sodium-chloride cotransporter
Source: J Biol Chem. 2021 Jun 24;297(2):100915. doi: 10.1016/j.jbc.2021.100915 (PMC8318901; doi:10.1016/j.jbc.2021.100915)
Supplement: Tables S1 and S2 and Figures S1–S9 [file mmc1.pdf]

SUPPLEMENTAL MATERIAL

High dietary potassium causes ubiquitin-dependent degradation of the kidney sodium-chloride cotransporter

Contents:

1. Supplemental Tables and Figures (page 2-12)

Supplemental Table 1. Physiological parameters of C57/bl6/J mice fed various diets 2
Supplemental Table 2. Overview of primer sets used for RTqPCR 3
Supplemental Figure 1. Effects of high K^+ on NCC 4
Supplemental Figure 2. Tubule viability analysis 5
Supplemental Figure 3. Effects of K^+ on ENaC 6
Supplemental Figure 4. Cellular ubiquitylation events are modulated by K^+ 7
Supplemental Figure 5. High K+ effects on PP1α occur in presence of aldosterone 8
Supplemental Figure 6. Immunoprecipitation of NCC, PP1α and Hsc70 9
Supplemental Figure 7. Effect of fostriecin on phosphorylated Gsk3β 10
Supplemental Figure 8. Effects of K^+ on I-1 11
Supplemental Figure 9. Characterization of mouse monoclonal anti-NCC antibody 12

Supplemental Table 1. Physiological parameters of C57/bl6/J mice fed various diets

	Control	High K
Bodyweight (g)	24.4 ± 0.5	24.4 ± 0.4
urine (ml/20 g BW)	1.6 ± 0.21	8.2 ± 0.5 *
dry food (g/20 g BW)	2.7 ± 0.1	2.9 ± 0.1
total water intake (ml/20 g BW)	4.3 ± 0.6	11.8 ± 0.6 *
Urine osmolalilty (mOsm)	1800 ± 139	819 ± 15 *
Osmolar excretion (mOsm/24hrs/20 g BW)	2755 ± 174	6695 ± 421*
Plasma sodium (mmol/l)	136.0 ± 1.7	140.7 ± 1.3
Plasma potassium (mmol/l)	4.9 ± 0.2	5.0 ± 0.1
Plasma chloride (mmol/l)	103.9 ± 1.7	97.0 ± 1.0 *
Plasma urea (mmol/l)	7.4 ± 0.3	6.9 ± 0.3
Plasma creatinine (µmol/l)	9.3 ± 0.9	11.2 ± 0.8
Urinary sodium concentration (mmol/l)	193 ± 14	46 ± 3 *
Urinary potassium concentration (mmol/l)	115 ± 7	257 ± 9 *
Urinary chloride concentration (mmol/l)	211 ± 17	54 ± 3 *
Urinary urea concentration (mmol/l)	939 ± 79	213 ± 8 *
Urinary creatinine concentration (µmol/l)	2277 ± 339	527 ± 28 *
Urinary sodium excretion (µmol/24 hrs)	330 ± 18	378 ± 26
Urinary potassium excretion (µmol/24 hrs)	196 ± 13	2120 ± 124*
Urinary chloride excretion(µmol/24 hrs)	362 ± 20	445 ± 28*
Urinary urea excretion (µmol/24 hrs)	1587 ± 84	1739 ± 126
Urinary creatinine excretion (nmol/24 hrs)	3900 ± 336	4128 ± 184
Creatinine clearance (ml/24hrs)	392 ± 56	342 ± 29
Plasma aldosterone (pg/ml)	104 ± 19	659 ± 234 *

* $p<0.05$ compared to control

Supplemental Table 2. Overview of primer sets used for RTqPCR

Gene (protein)	Forward primer	Reverse primer
Slc12a3 (NCC)	CATCAAGAACTACCGCCCCC	CTCTGCTTGCCAGGTCCAAT
Slc12a3 (NCC)	GCCTCCATCACCAACTCACC	AGTTCACCTCTGGCTTCTTGTA
Hspa8 (Hsc70)	GACAAGGGCCGCTTGAGTAA	GAAGGCATAGGACTCCAGTGAG
Hspa8 (Hsc70)	CCCGATGAAGCTGTTGCCTA	TGGTATTGCGCTTGATGAGGA
Ppp1ca (PP1α)	GCATCAACCGCATTTATGGCT	CGTCTGTGGGCCGCATAATA
Ppp1ca (PP1α)	ATGAGTGTGCCAGCATCAACC	TGGATTGCAAGTCTGGAGACAG
Ppp1cb (PP1β)	TGTGGCGAGTTTGACAATGC	GGATTAGCTGTTCGAGGCGG
Ppp1cb (PP1β)	TGCTGGTGGTATGATGAGTGT	TCTTCGGTGGATTAGCTGTTCG
Ppp1cc (PP1γ)	TGCGCCAGCATCAATAGGAT	ATCTTCTCGTCCACGATGGC
Ppp1cc (PP1α)	TCGACAGCATCATCCAACGG	GATCTCCCGAGACTTCAGGC
18S	GGATCCATTGGAGGGCAAGT	ACGAGCTTTTTAACTGCAGCAA

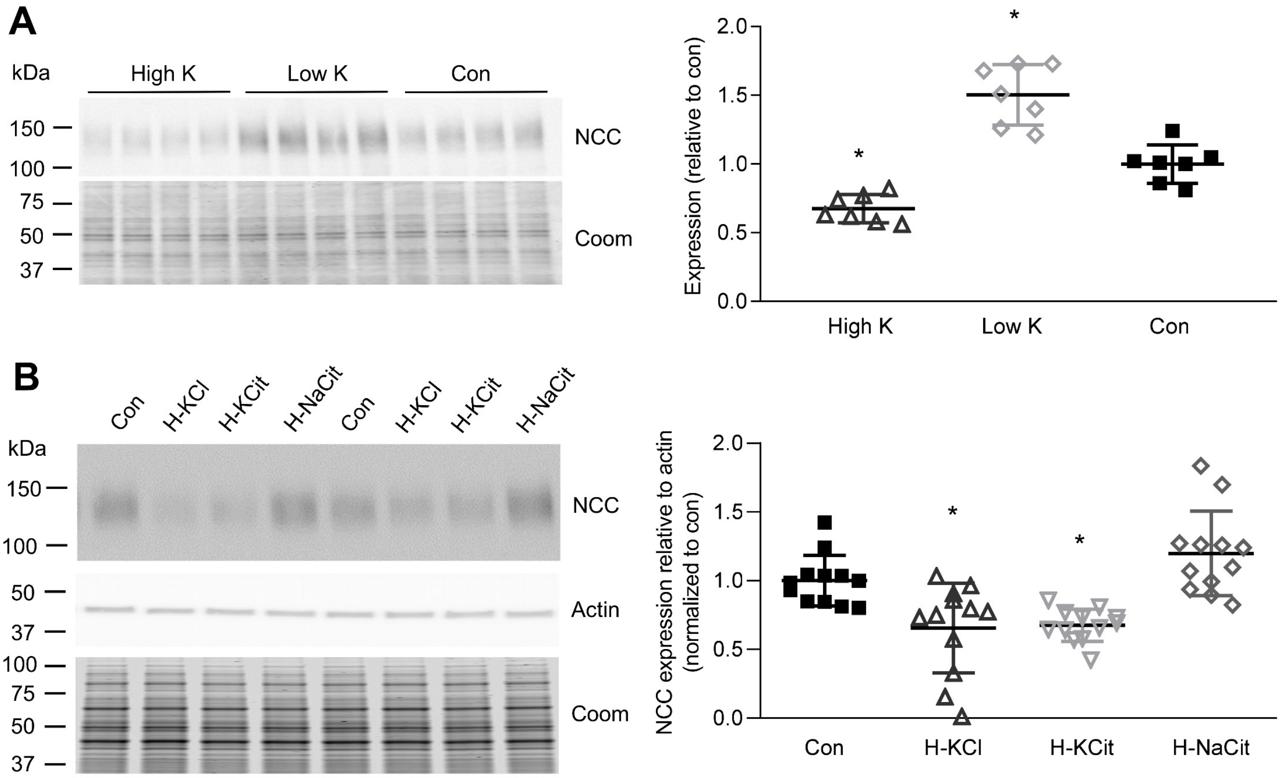

Supplemental Figure 1. Effects of high K^+ on NCC. A) Freshly isolated cortical tubules were incubated in medium with either low K^+ (0.5 mM K^+), control (3.5 mM K^+) or high K^+ (8.0 mM K^+) for 24 h. NaCl was used to adjust the chloride to equivalent concentrations in each of the media. Tubules were harvested and subjected to immunoblotting for NCC. Summary data show normalized signal intensity relative to control (n=7). **P*<0.05 compared to control media. B) Tubule suspensions were incubated in control medium (3.5 mM K^+), high KCl (8 mM K^+), high K^+-citrate (8 mM K^+) or high Na-citrate (3.5 mM K^+ with an additional 4.5 mM Na^+). Tubules were harvested and subjected to immunoblotting for NCC and actin. Summary data show normalized signal intensity relative to control (n=12). **P*<0.05 compared to control media. A+B: shown are mean values ± SD. Comparisons were performed using a one-way ANOVA followed by a Dunnett multiple comparison test. Coom = Coomassie blue staining.

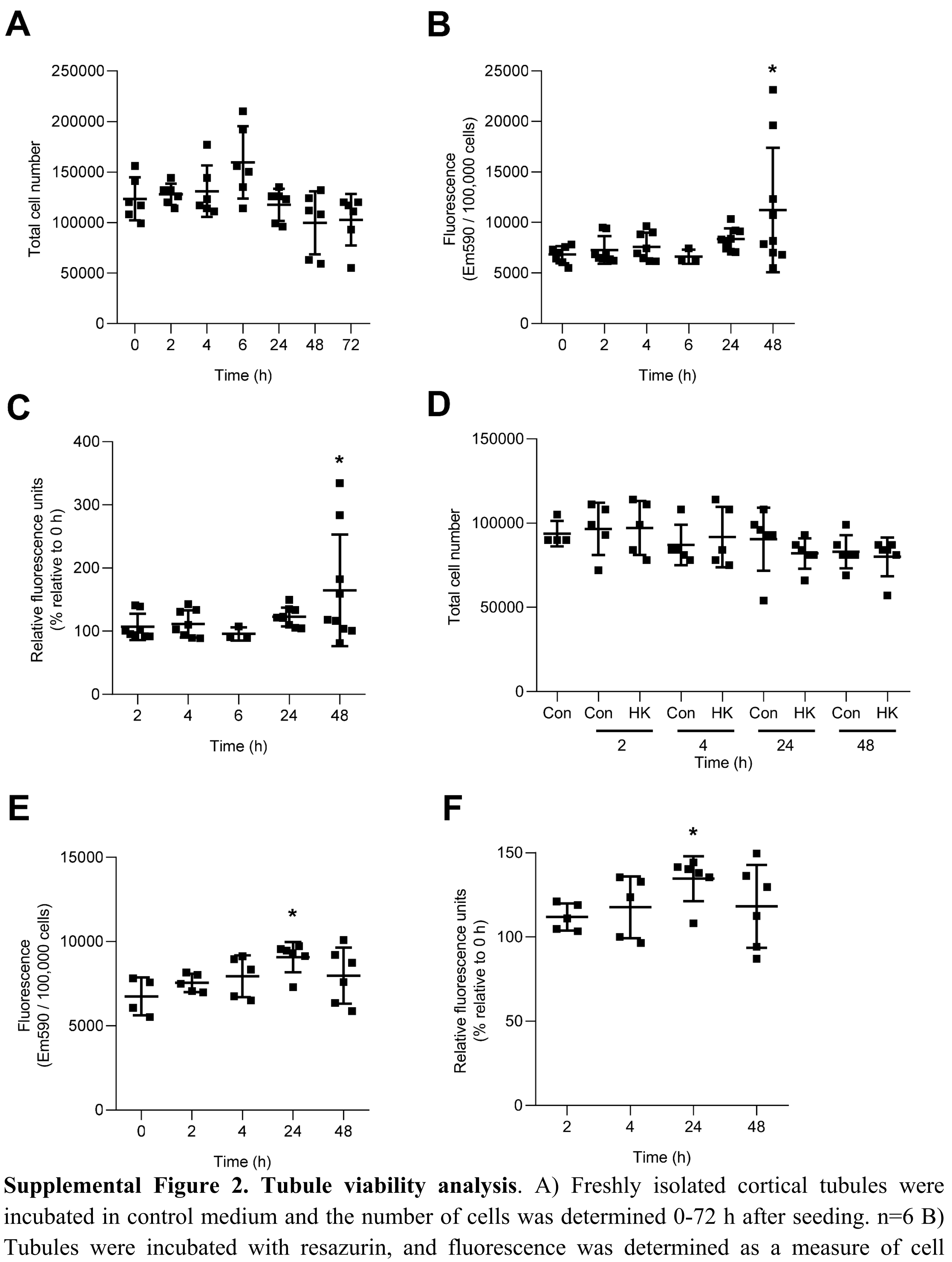

Supplemental Figure 2. Tubule viability analysis. A) Freshly isolated cortical tubules were incubated in control medium and the number of cells was determined 0-72 h after seeding. n=6 B) Tubules were incubated with resazurin, and fluorescence was determined as a measure of cell viability. n=3-9. C) Values obtained in “B” shown as relative to 0 h. D) Freshly isolated cortical tubules were incubated in control or high K^+ medium and the number of cells was determined 0-48 h after seeding. n=4-6. E) Tubules grown in high K^+ medium were incubated with resazurin, and fluorescence was determined as a measure of cell viability. n=4-6. F) Values obtained in “E” shown as relative to 0 h. Significant differences ($P<0.05$) to 0 h are indicated by *. A+F: shown are mean values ± SD. Comparisons were performed using a one-way ANOVA followed by a Dunnett multiple comparison test.

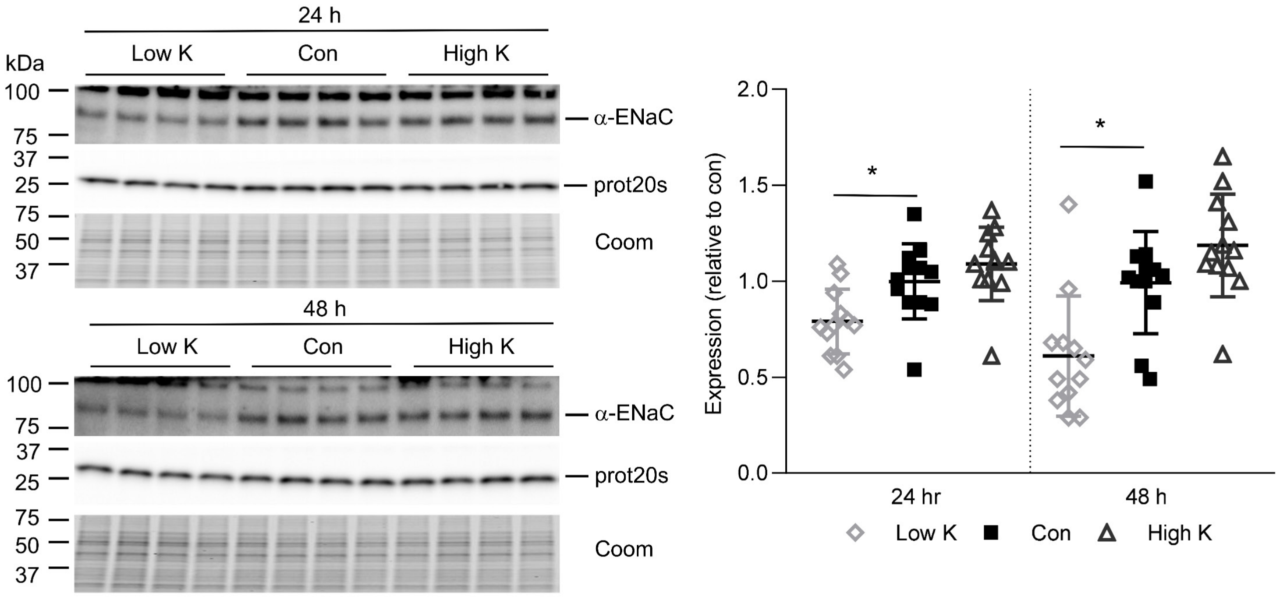

Supplemental Figure 3. Effects of K$^+$ on alpha (α) ENaC. A) Freshly isolated cortical tubules were incubated in medium with either low K$^+$ (0.5 mM K$^+$), control (3.5 mM K$^+$) or high K$^+$ (8.0 mM K$^+$) for 24 and 48 h. Tubules were harvested and subjected to immunoblotting for α-ENaC. Summary data show normalized signal intensity relative to control (n=9). **P*<0.05 compared to control media. Comparisons were performed using a one-way ANOVA followed by a Dunnett multiple comparison test. Coom = Coomassie blue staining.

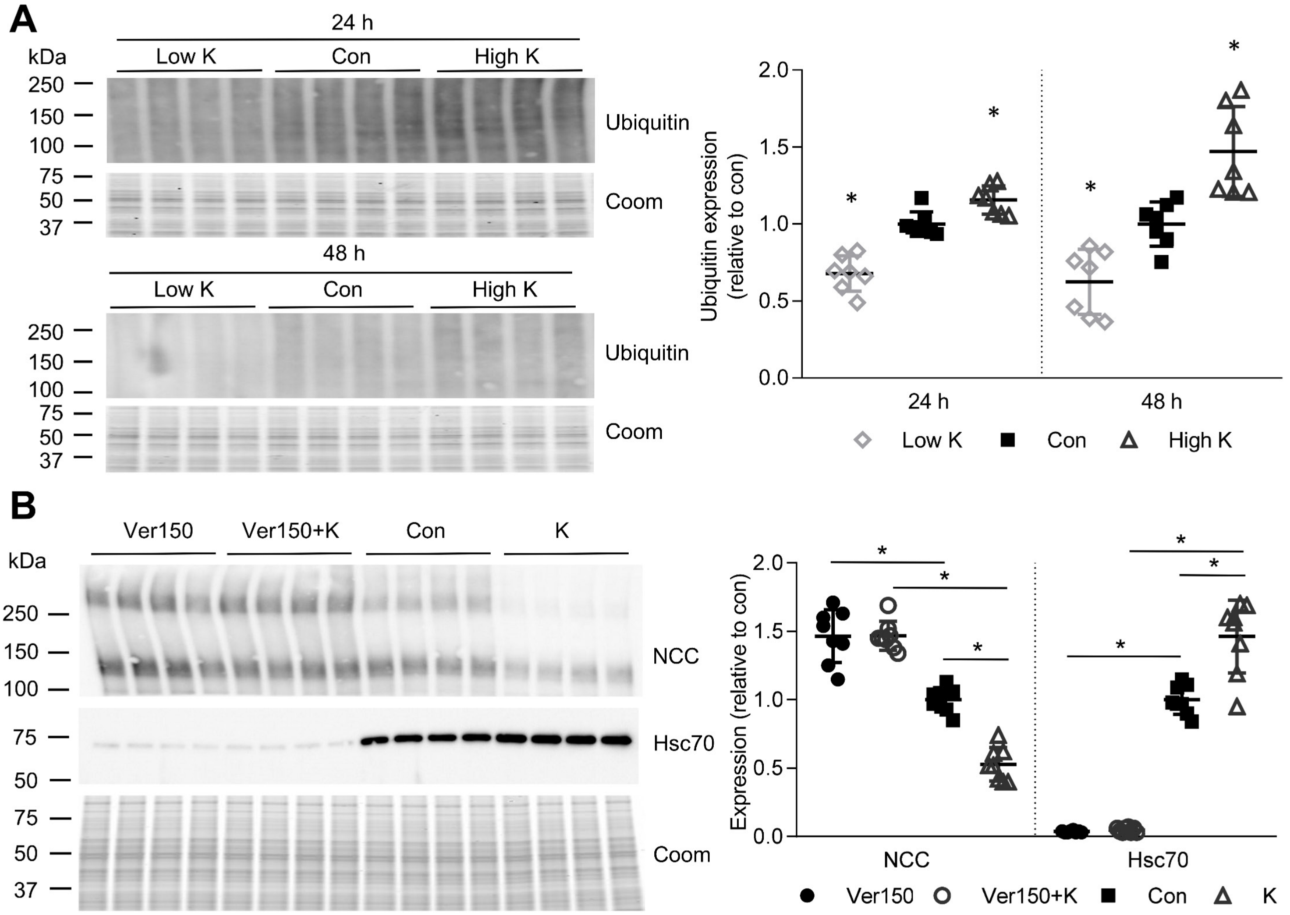

Supplemental Figure 4. Cellular ubiquitylation events are modulated by K^+. A) Freshly isolated cortical tubules were incubated in either low K^+ (0.5 mM K^+), control (Con, 3.5 mM K^+) or high K^+ (8.0 mM K^+) medium and ubiquitin levels assessed after 24 and 48 h. Summary data (mean ± SD) show normalized signal intensity relative to control (n=7). **P*<0.05 compared to control media. Comparisons were performed using a one-way ANOVA followed by a Dunnett multiple comparison test. The same samples were used for blotting as for generating the data for Fig. 1A, hence the coomassie gels are the same. B) Freshly isolated cortical tubules were incubated in control (Con, 3.5 mM K^+) or high K^+ medium (K, 8.0 mM K^+) for 48 h, with or without 150 µM of the Hsp70/Hsc70 blocker Ver-155008 (Ver), and NCC and Hsc70 levels assessed by immunoblotting. Summary data (mean ± SD) show normalized band densities relative to control and significant differences are indicated (**P*<0.05). Comparisons were performed using a two-way ANOVA followed by a Tukey multiple comparison test. Coom = Coomassie blue staining.

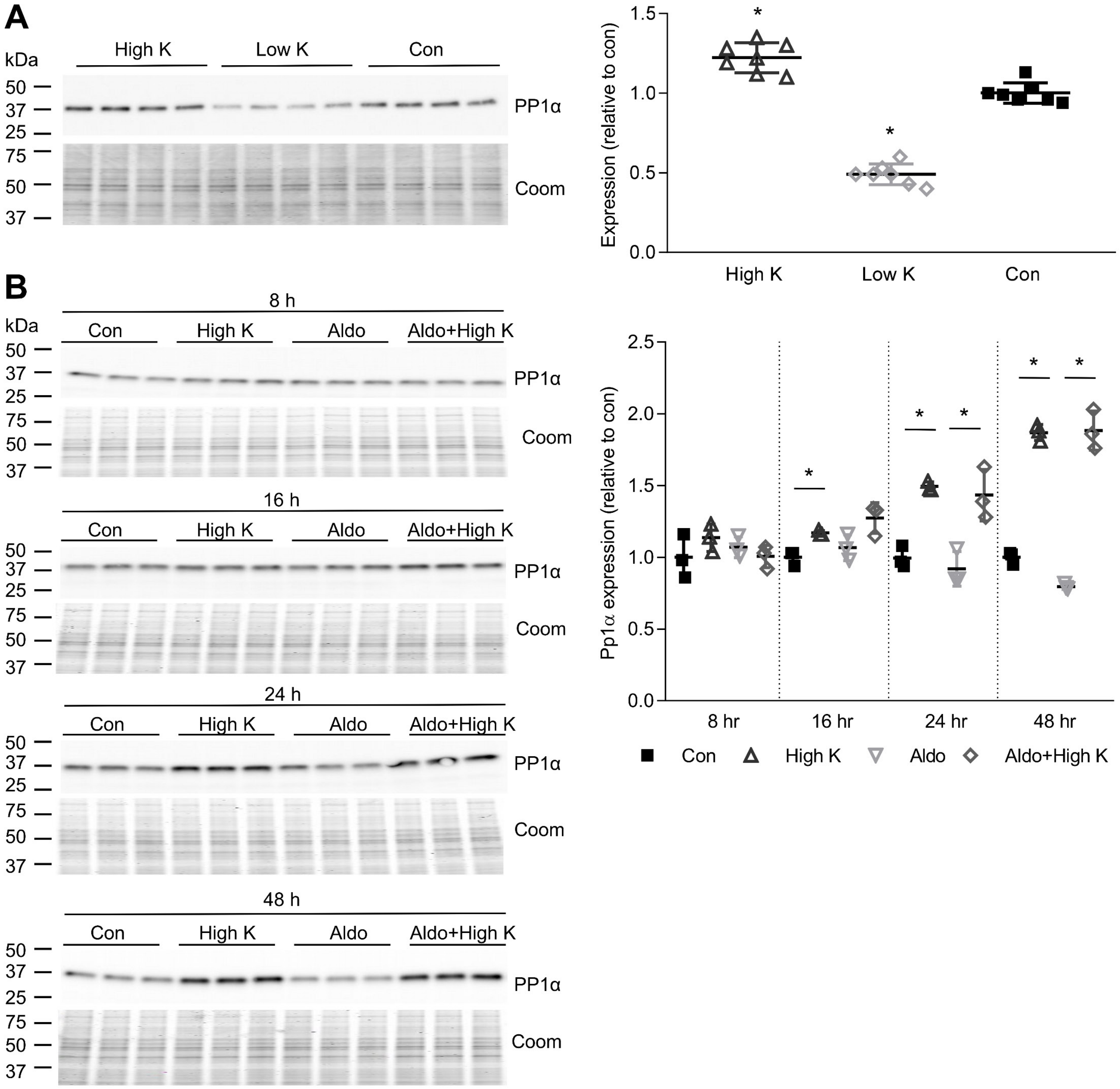

Supplemental Figure 5. High K$^+$ effects on PP1α occur in presence of aldosterone. A) Freshly isolated cortical tubules were incubated in medium with either low K$^+$ (0.5 mM K$^+$), control (3.5 mM K$^+$) or high K$^+$ (8.0 mM K$^+$) for 24 h. NaCl was used to adjust the chloride to equivalent concentrations in each of the media. Tubules were harvested and subjected to immunoblotting for PP1α. The same samples were used for blotting as for generating the data for Suppl. Fig 1A, hence the coomassie gels are the same. Summary data show normalized signal intensity (mean values ± SD) relative to control (n=7). **P*<0.05 compared to control media. Comparisons were performed using a one-way ANOVA followed by a Dunnett multiple comparison test. B) Tubules were incubated in either control (Con, 3.5 mM K$^+$) or high K$^+$ (8.0 mM K$^+$) medium with or without 10 nM aldosterone. Tubules were harvested after A) 8 h, B) 16 h, C) 24 h and D) 48 h, and subjected to immunoblotting for PP1α. Summary data show normalized signal intensity (mean values ± SD) relative to control (n=3) and significant differences are indicated (**P*<0.05). Comparisons were performed using a two-way ANOVA followed by a Tukey multiple comparison test. Coom = Coomassie blue staining. The same samples were used for blotting as for generating the data for Fig. 2A-D, hence the coomassie gels are the same.

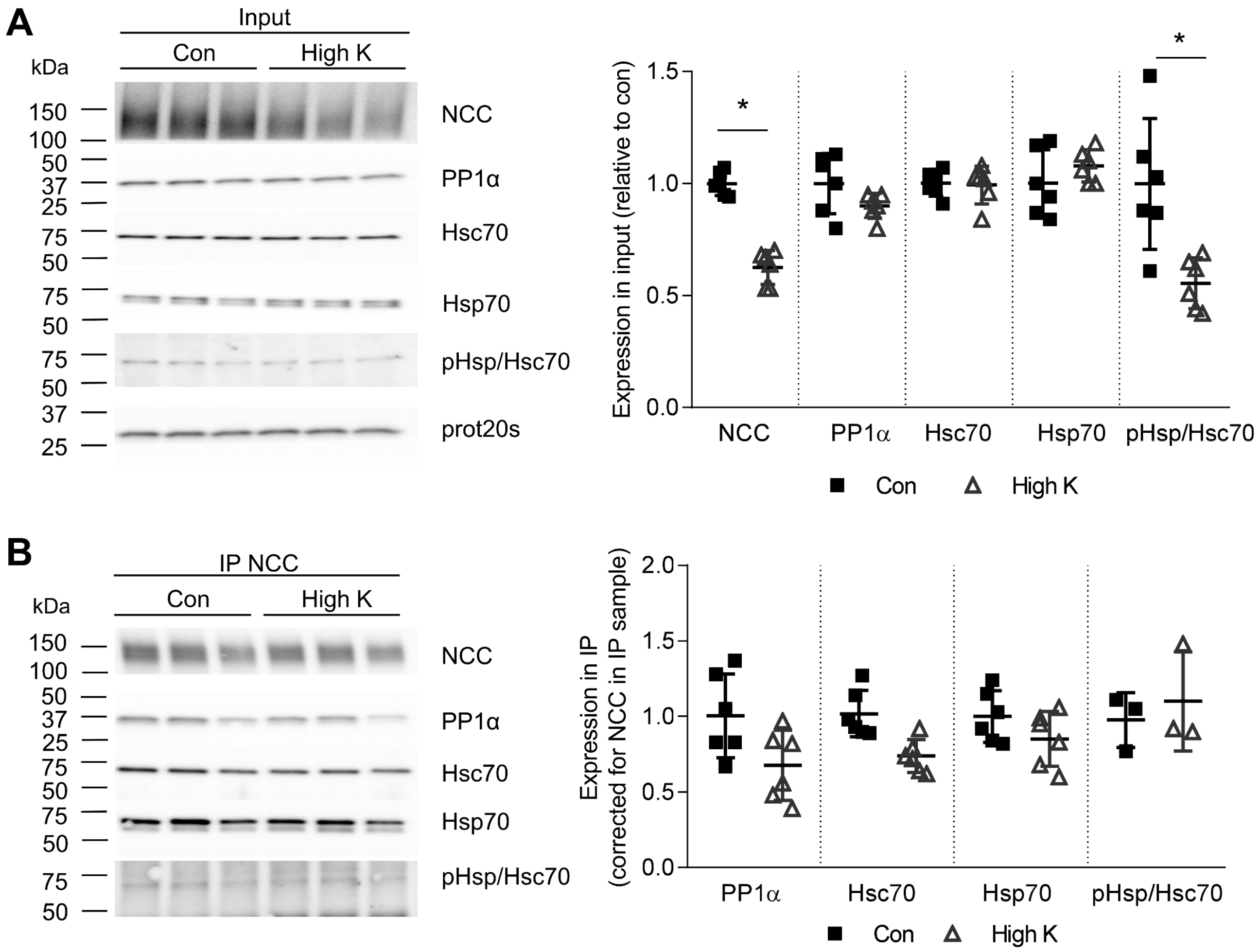

Supplemental Figure 6. Immunoprecipitation of NCC, PP1α and Hsc70. Tubules were incubated in control (3.5 mM) or high K^+ medium and lysates were subjected to immunoprecipition using an NCC antibody. Input samples (A) and immunoprecipitated samples (B) were assessed for levels of NCC, PP1α, Hsc70, Hsp70 and phosphorylated Hsp/Hsc70 (pHsp/Hsc70). Summary data (mean values ± SD) show densities relative to control. IP=immunoprecipitation. Significant differences are indicated (**P*<0.05) (n=6). Comparisons were performed using an unpaired students *t*-test.

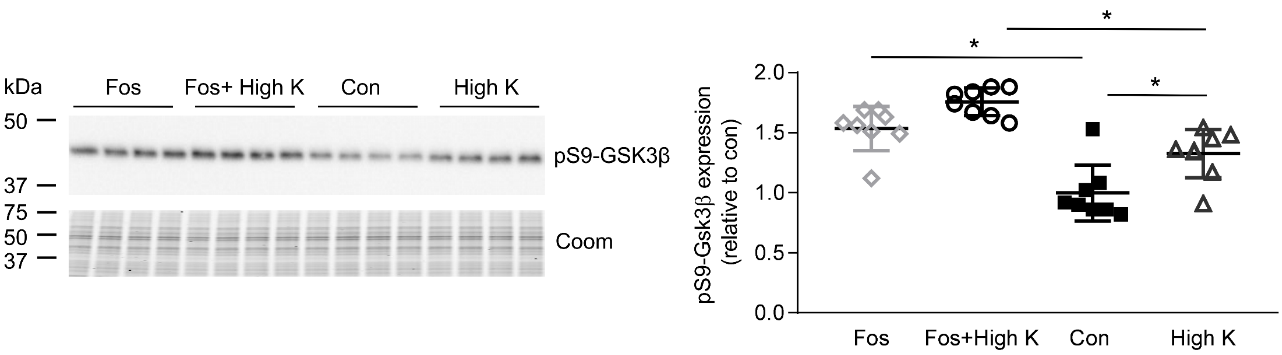

Supplemental Figure 7. Effect of fostriecin on phosphorylated Gsk3β. Tubules were incubated in control (Con, 3.5 mM K^+) or high K^+ medium (K, 8.0 mM K^+) for 24 h, with or without 1.5 µM fostriecin (Fos), and levels of phosphorylated Gsk3β (pS9-GSK3β), a known PP2A target were assessed by immunoblotting. Summary data (mean values ± SD) show normalized band densities relative to control (n=8) and significant differences are indicated (*P<0.05). Comparisons were performed using a two-way ANOVA followed by a Tukey multiple comparison test. Coom = Coomassie blue staining. The same samples were used for blotting as for generating the data for Fig. 7G, hence the coomassie gels are the same.

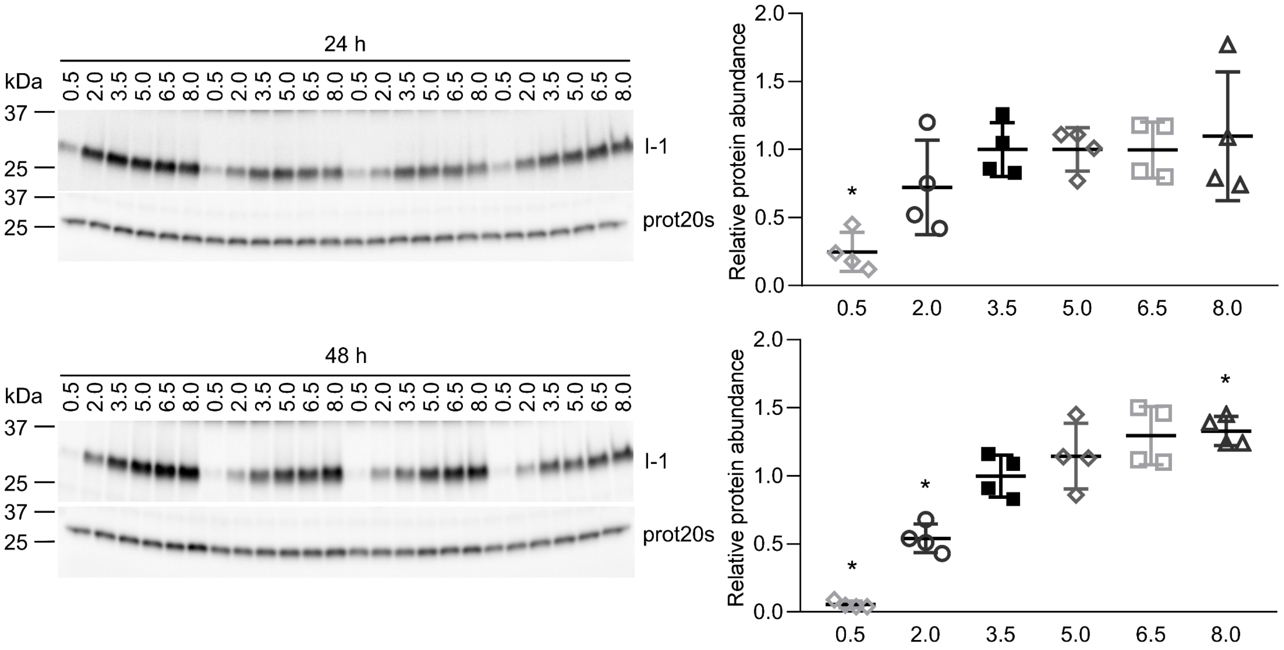

Supplemental Figure 8. Effects of K^+ on I-1. Isolated renal cortical tubules were incubated in medium with varying K^+ concentrations and I-1 protein levels assessed after 24 and 48 h. Summary data of I-1 normalized band densities relative to 3.5 mM K^+ control media (mean ± SD, n=4). *$P<0.05$ compared to control media.

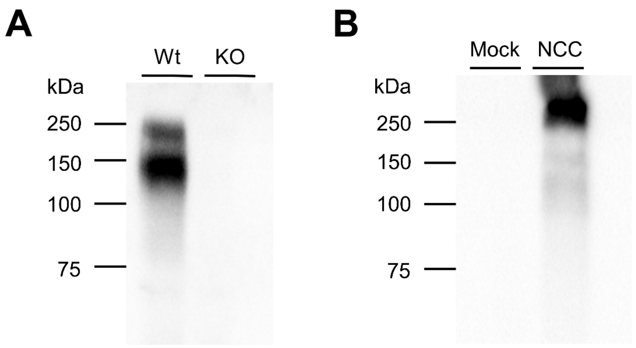

Supplemental Figure 9. Characterization of mouse monoclonal anti-NCC antibody. Immunoblotting of NCC using a mouse monoclonal anti-NCC antibody. A) Immunoblotting of kidney tissue obtained from wild-type (Wt) and NCC-deficient (KO) mice. B) Immunoblotting of HEK293 cells transfected with human SLC12A3 (NCC) or mock.
